# A novel murine model to study the impact of maternal depression and antidepressant treatment on biobehavioral functions in the offspring

**DOI:** 10.1038/s41380-021-01145-7

**Published:** 2021-05-17

**Authors:** Joseph Scarborough, Flavia S. Mueller, Ulrike Weber-Stadlbauer, Daniele Mattei, Lennart Opitz, Annamaria Cattaneo, Juliet Richetto

**Affiliations:** 1 Institute of Pharmacology and Toxicology, University of Zurich-Vetsuisse, Zurich, Switzerland; 2 Neuroscience Center Zurich, University of Zurich and ETH Zurich, Zurich, Switzerland; 3 Department of Neurology, Icahn School of Medicine at Mount Sinai Hospital, New York, NY USA; 4 Functional Genomics Center Zurich, Swiss Federal Institute of Technology and University of Zurich, Zurich, Switzerland; 5 Biological Psychiatry Unit, IRCCS Fatebenefratelli San Giovanni di Dio, Brescia, Italy; 6 Department of Pharmacological and Biomolecular Sciences, University of Milan, Milan, Italy

**Keywords:** Neuroscience, Molecular biology

## Abstract

Antenatal psychopathology negatively affects obstetric outcomes and exerts long-term consequences on the offspring’s wellbeing and mental health. However, the precise mechanisms underlying these associations remain largely unknown. Here, we present a novel model system in mice that allows for experimental investigations into the effects of antenatal depression-like psychopathology and for evaluating the influence of maternal pharmacological treatments on long-term outcomes in the offspring. This model system in based on rearing nulliparous female mice in social isolation prior to mating, leading to a depressive-like state that is initiated before and continued throughout pregnancy. Using this model, we show that the maternal depressive-like state induced by social isolation can be partially rescued by chronic treatment with the selective serotonin reuptake inhibitor, fluoxetine (FLX). Moreover, we identify numerous and partly sex-dependent behavioral and molecular abnormalities, including increased anxiety-like behavior, cognitive impairments and alterations of the amygdalar transcriptome, in offspring born to socially isolated mothers relative to offspring born to mothers that were maintained in social groups prior to conception. We also found that maternal FLX treatment was effective in preventing some of the behavioral and molecular abnormalities emerging in offspring born to socially isolated mothers. Taken together, our findings suggest that the presence of a depressive-like state during preconception and pregnancy has sex-dependent consequences on brain and behavioral functions in the offspring. At the same time, our study highlights that FLX treatment in dams with a depression-like state can prevent abnormal behavioral development in the offspring.

## Introduction

Pregnancy is a delicate period that relies on a balance between personal, family-related and employment-related issues. In instances where this balance is not attainable, pregnancy can become a stress-inducing situation, which in turn compromises the mother and offspring’s wellbeing [1]. In high-income countries, ~5–15% of pregnant women meet the criteria for depression and/or clinical anxiety [2–4], and these rates are even higher in low- and middle- income countries [4, 5]. Consequently, the use of antidepressant drugs (ADDs) during pregnancy has greatly increased, and is expected to escalate given the overall rise of depression worldwide [6, 7].

The most commonly prescribed ADDs are selective serotonin reuptake inhibitors (SSRIs), which have been considered moderately safe for antenatal use [6, 8, 9]. SSRIs act by inhibiting the serotonin transporter, thus blocking the reabsorption of serotonin by presynaptic neurons and increasing its availability in the synaptic cleft [10]. Importantly, at least some SSRIs administered in pregnancy can cross the placenta and are excreted in breast milk, thus reaching the fetus and the newborn, respectively [11, 12]. Since the serotoninergic system and its downstream effectors are critical for normal neurodevelopment [13, 14], it is plausible that manipulating this system during fetal and perinatal life by exposure to SSRIs may have the potential to alter brain development.

Some recent human studies seem to suggest that the use of SSRIs during pregnancy could pose a risk for the developmental health of the offspring [7], increasing, for example, the risk of preterm delivery [15, 16], low birth weight and cardiac defects [15, 17]; the development of mental illness [18–20]; and alterations in the hypothalamic-pituitary-adrenal (HPA) axis and the serotoninergic system [21, 22]. However, the results of human studies are equivocal, with other reports finding no such associations [23], and few are graded as high-quality evidence [24]. Moreover, they are often associated with multiple confounding factors, in primis the underlying maternal psychopathology. Indeed, SSRI treatment may just be a marker of the severity of the depressive symptoms in the mother, which may be the actual cause of the alterations reported in the offspring [25]. Maternal mood disorders per se are, in fact, associated with a variety of detrimental consequences for the newborn [26–29], and numerous reports suggest that untreated prenatal depression is associated with increased infant morbidity and neurodevelopmental abnormalities [4, 27, 30, 31]. Thus, despite the possible risks associated with SSRI treatment during pregnancy, these compounds continue to be critically important for the wellbeing of depressed mothers, and discontinuation of therapy is not recommended [32]. In view of these findings, further research is warranted in this clinically important domain, with emphasis on discerning the relative effects of SSRI treatment from maternal psychopathology.

One way to overcome the difficulties of assessing the effects of developmental exposure to SSRIs and maternal depression is through the use of animal models, which allow for detailed investigations of the short- and long-term consequences of maternal stress and/or SSRI treatment under stringent experimental conditions. While a plethora of animal models of prenatal stress exist [33], the large majority of studies assessing developmental SSRI exposure did not assess the effects of these drugs in a model of antenatal psychopathology, but rather in normal pregnant animals [23]. The latter may, however, undermine the relevance and translatability to the clinical context of maternal depression treated with ADDs [34]. Indeed, the very presence of maternal depression, a disorder characterized by an underlying behavioral and neurobiological pathology [31], could influence the effects of maternal SSRI exposure on the offspring, thus rendering healthy pregnant dams inadequate to model the pathological conditions in humans [23].

Exposure to sub-chronic or chronic physical stressors during pregnancy is frequently used to model the effects of prenatal stress (with or without concomitant SSRI treatment) as a proxy of human prenatal depression in animals [23, 35–40]. A possible caveat of this approach is that it mostly relies on exposing pregnant animals to physical stressors, such as restraint stress or foot-shook, in order to induce a temporary depressive-like state during pregnancy [33]. However, since depression in humans is believed to be elicited by social stress rather than physical stress [41–43], these models readily fail to account for the social stress component of depression. Moreover, the strongest risk factor for depression during pregnancy is a history of recent depression [44, 45], and the most clinically challenging cases are those in which depression is present before and/or throughout the whole of pregnancy, as opposed to post-partum or during specific stages of pregnancy only [46, 47]. Thus, the application of stress confined to specific phases of the gestational window may be insufficient to model the complete complex spectrum of antenatal maternal depression.

Here, we present a new murine model to study the consequences of antenatal maternal depression and SSRI treatment in the offspring, which encompasses both the social stress component of depression and its presence before and throughout gestation. The model is based on the well-known social isolation rearing (SIR) paradigm, which has been shown to mimic a variety of symptoms common to anxiety and depression, such as impaired reward processing, anxiety-like behaviors, metabolic alterations and serotoninergic pathology [48–55]. Yet, the effects of SIR have not yet been investigated in the context of models of antenatal maternal depression, including examinations of its effects on the offspring. In the present study, we applied the SIR paradigm to nulliparous female mice prior to mating in order to induce a depression-like state that is initiated before and continued throughout pregnancy. Using this model, we show that the maternal depression-like state induced by SIR can be partially rescued by chronic treatment with the SSRI, fluoxetine (FLX). Moreover, we identify a variety of behavioral and transcriptomic abnormalities in male and female offspring born to socially isolated mothers, which can partly be prevented by maternal FLX treatment.

## Methods

### Animals

C57BL6/N mice (Charles River Laboratories, Sulzfeld, Germany) were used throughout the study and maintained in an animal vivarium as described in Supplementary 1.

### Social isolation paradigm

**Fig. 1:**
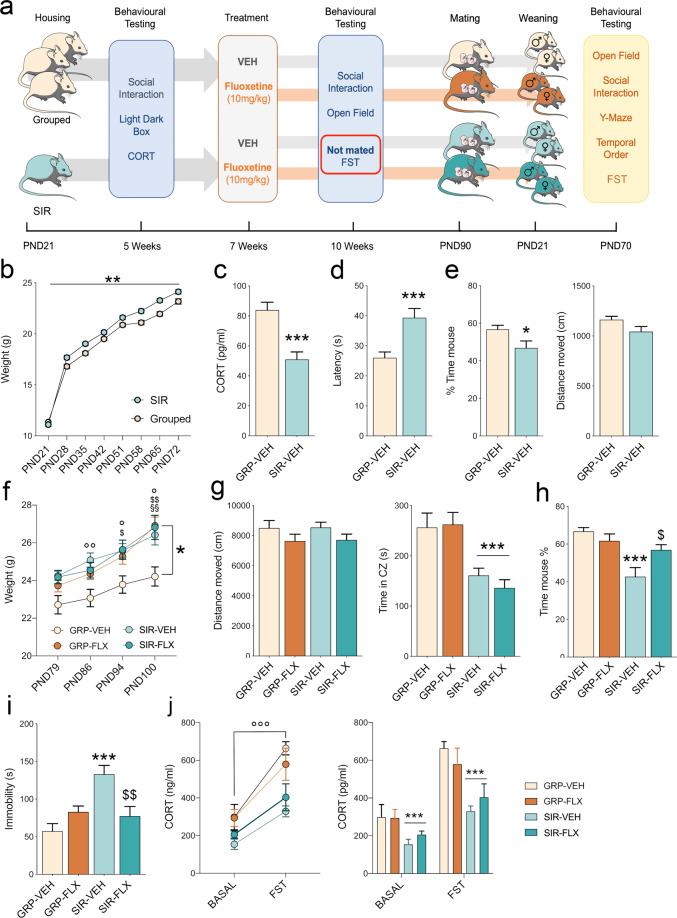
Experimental paradigm and behavioral readouts in SIR and GRP-housed female mice. Physiological and behavioral effects of SIR in female mice prior to conception. **a** Graphical representation of the experimental design. C57BL6/N female mice were housed in social isolation or in groups from PND21 onwards. After 5 weeks of social isolation or group housing, the animals were subjected to behavioral testing in the light-dark box test and social interaction test as well as to basal CORT measurements. After 7 weeks of social isolation or group housing, each group of SIR and GRP animals were split into two subgroups and treated with either vehicle (drinking water) or FLX (10 mg/kg). After 10 weeks, the animals were subjected to behavioral testing in the open field test and the social interaction test. A subgroup of animals was subjected to the forced swim test and to basal and stress-induced CORT measurements. These animals were excluded from mating. Weight gain was assessed weekly during the whole experimental timeline. The animals were then exposed to a timed mating procedure and their offspring were left undisturbed until adulthood. Maternal FLX treatment was continued throughout mating, pregnancy and the offspring’s pre-weaning period. Adult offspring were subjected to behavioral testing in the open field test, the social interaction test, the Y-maze test of spatial recognition memory, the temporal order test and the forced swim test (FST). Weight gain was monitored throughout development, while basal and stress-induced levels of CORT were measured before and after the FST, respectively. **b** Weight gain in SIR and GRP females during the first 7 weeks of isolation- or group-housing. **p < 0.01, reflecting the main effect of housing. **c** Basal plasma levels of CORT in SIR and GRP females at the 5-weeks testing timepoint; ***p < 0.001. **d** Latency to shuttle to the bright compartment of the light-dark box testing apparatus; ***p < 0.001. **e** Percent exploration time of an unfamiliar mouse compared to a dummy object and total distance moved during the social interaction test at the 5 weeks testing timepoint; *p < 0.05. **f** Weight gain in SIR-VEH, SIR-FLX, GRP-VEH and GRP-FLX animals in the three weeks following the start of FLX treatment (SIR-VEH *vs* GRP-VEH at PND86: °°*p* < 0.01; SIR-VEH *vs* GRP-VEH at PND94: °*p* < 0.05; SIR-FLX *vs* GRP-VEH at PND94: ^$^*p* < 0.05; SIR-VEH *vs* GRP-VEH at PND100: °*p* < 0.05; SIR-FLX *vs* GRP^-^VEH at PND100: ^$$^*p* < 0.01; GRP-FLX *vs* GRP-VEH at PND100: ^§§^*p* < 0.01). **g** Total distance moved and time spent in the center zone in the open field test at the 10 weeks testing timepoint; ***p < 0.001, reflecting the main effect of SIR. **h** Percent exploration time of an unfamiliar mouse compared to a dummy object and total distance moved during the social interaction test at the 10 weeks testing timepoint; ***p < 0.001 between SIR-VEH and GRP-VEH; ^$^*p* < 0.05 between SIR-VEH and SIR-FLX. **i** Time spent immobile in the FST test at the 10 weeks testing timepoint. ^$$^*p* < 0.01 between SIR-VEH and SIR-FLX; ***p < 0.001 between SIR-VEH and GRP-VEH. **j** Basal and stress-induced levels of CORT. °°°*p* < 0.001, reflecting the main effect of testing condition; ***p < 0.001, reflecting the main effect of housing. *N* = 40 mice per group in **b**–**e**, *N* = 20 animals per group in **f**–**h**, and *N* = 10 animals per group in **i**–**j**. All values represent means ± s.e.m.

Female mice at postnatal day (PND) 21 were either housed individually (*N* = 40) or group housed (*N* = 40) for 10 weeks until they were subjected to selected behavioral testing followed by a timed mating procedure (Supplementary 1). A schematic representation of the experimental paradigm, animal cohorts and numbers are provided in Fig. 1a and in Supplementary Tables 1 and 2, respectively.

### Fluoxetine treatment

FLX treatment commenced after 7 weeks of social isolation or group housing (Fig. 1a) and continued throughout mating, pregnancy and weaning of the offspring, and thus lasted for a total of 10 weeks. FLX (10 mg/kg) was administered in the drinking water using protocols established and validated before [56] (Supplementary 1).

### Behavioral testing

Behavioral testing was conducted both in socially isolated and group-housed female animals, and in their offspring (Fig. 1a). In the dams, behavioral testing was conducted after 5 and 10 weeks of the social isolation regimen in order to ascertain anxiety- and depressive-like behaviors (Fig. 1a and Supplementary 1).

The offspring were tested once they reached adulthood (PND 70 onwards) in the above-mentioned behavioral tests. In addition, they were subjected to a Y-maze spatial recognition test and a temporal order memory test to assess their cognitive functions. A detailed description of each test, the testing order, and number of animals is provided in Supplementary 1 and in Supplementary Tables 1 and 2.

### Corticosterone measurements

Blood samples were taken from the tail vein as described previously [57] and in Supplementary 1. Plasma was collected after centrifugation at 10,000 × *g* at 4 °C for 10 min and then stored at −20 °C until CORT analysis was performed. CORT was measured using the DirectX corticosterone enzyme immunoassay kit (Arbor Assays, Ann Arbor, MI, USA) following the manufacturer’s instructions.

### Next-generation RNA sequencing

Transcriptomic analysis was assessed in the amygdala using next-generation RNA-seq, gene network analysis and qPCR as described in Supplementary 1 and Supplementary Table 3. RNA was extracted (Lexogen SPLIT kit) and RNA-seq libraries were prepared using the Universal Plus mRNA kit (Nugen). The amygdala was chosen based on its critical involvement in regulating emotional processing and affective behaviors [58, 59].

### Statistical analysis

All data met the assumptions of normal distribution and equality of variance; and all data were analyzed using parametric analysis of variance (ANOVA) or student’s *t* Test. Whenever appropriate, ANOVAs were followed by Tukey’s post-hoc test to control for multiple comparisons. Main effects of ANOVA are described in the main text, while post-hoc comparisons are reported in the figure legends. All statistical analyses were performed using SPSS Statistics (version 22.0, IBM, Armonk, NY, USA) and Prism (version 7.0; GraphPad Software, La Jolla, CA, USA). Statistical significance was set at *p* < 0.05. A detailed description of each statistical analysis is provided in Supplementary 1.

## Results

### Effects of SIR and FLX treatment during preconception

First, we ascertained the physiological and behavioral effects of SIR in female mice prior to conception. In line with previous studies [50], SIR females gained more weight over time (main effect of housing: *F*_(1,78)_ = 11.661*, p* < 0.001; Fig. 1b) and displayed significantly lower basal CORT plasma levels (*t*_(78)_ = 4.571, *p* < 0.0001, Fig. 1c) as compared to group-housed (GRP) animals 5 weeks after SIR. At this time interval, SIR females also displayed increased anxiety-like behavior in the light-dark box test, as evident by the increased latency to shuttle from the dark to the bright compartment of the testing apparatus (*t*_(78)_ = 3.683, *p* < 0.001, Fig. 1d). Moreover, compared to GRP mice, SIR mice showed decreased sociability in the social interaction test (*t*_(78)_ = 2.288, *p* < 0.05, Fig. 1e), which was not confounded by differences in locomotor activity (*t*_(78)_ = 1.927, *n.s*., Fig. 1e).

We next assessed whether chronic FLX treatment would influence the physiological and behavioral effects of SIR. To this end, half of the SIR and GRP mice were subjected to chronic FLX treatment at 7 weeks after isolation rearing or group housing, whereas the other half of mice was assigned to vehicle (VEH) treatment (Fig. 1a). As shown in Fig. 1f, FLX treatment did not mitigate the weight gain induced by SIR (main effect of housing: *F*_(1,44)_ = 6.507, *p* < 0.05) but increased the weight gain in GRP females (housing x treatment interaction: *F*_(1,44)_ = 4.010, *p* < 0.05). Likewise, FLX treatment did not normalize signs of innate anxiety-like behavior in the open field test, where VEH- and FLX-treated SIR mice similarly displayed a significant reduction (main effect of housing: *F*_(1,76)_ = 26.26, *p* < 0.0001) in the time spent in the center zone (Fig. 1g). FLX treatment, however, mitigated the social interaction deficit induced by SIR, leading to a significant interaction between housing and FLX treatment (*F*_(1,76)_ = 7.596, *p* < 0.01). As shown in Fig. 1h, SIR-VEH animals failed to show a preference towards an unfamiliar live mouse when compared to GRP-VEH animals, whereas FLX treatment in SIR animals normalized this deficit. Moreover, FLX treatment mitigated the increase in time spent immobile in the forced swim test displayed by SIR-VEH females, leading to a significant interaction between housing and FLX treatment (*F*_(1,36)_ = 14.33, *p* < 0.001).

Lastly, chronic treatment with FLX did not affect basal and stress-induced levels of CORT, which were reduced in SIR females regardless of treatment (main effect of housing: *F*_(1,36)_ = 22.20, *p* < 0.0001). GRP and SIR animals displayed a similar increase in plasma CORT after the forced swim test regardless of FLX treatment (main effect of test: *F*_(1,36)_ = 53.69, *p* < 0.0001), with both basal and stress-induced CORT being lower in SIR females compared to GRP females (Fig. 1j).

### Effects of maternal SIR and FLX treatment on behavior and cognition in the offspring

We next performed physiological and behavioral characterizations in the adult offspring born to VEH- or FLX-treated SIR and GRP females to explore whether maternal SIR and/or FLX treatment would induce lasting functional changes in the progeny. Neither maternal SIR, nor maternal FLX treatment, exerted significant effects on litter size, sex distribution and pup weight in male and female offspring at weaning (PND21) (Supplementary Figs. S1 and S2). However, maternal SIR and FLX treatment both affected weight gain in male but not female offspring (Supplementary Fig. S3).

**Fig. 2:**
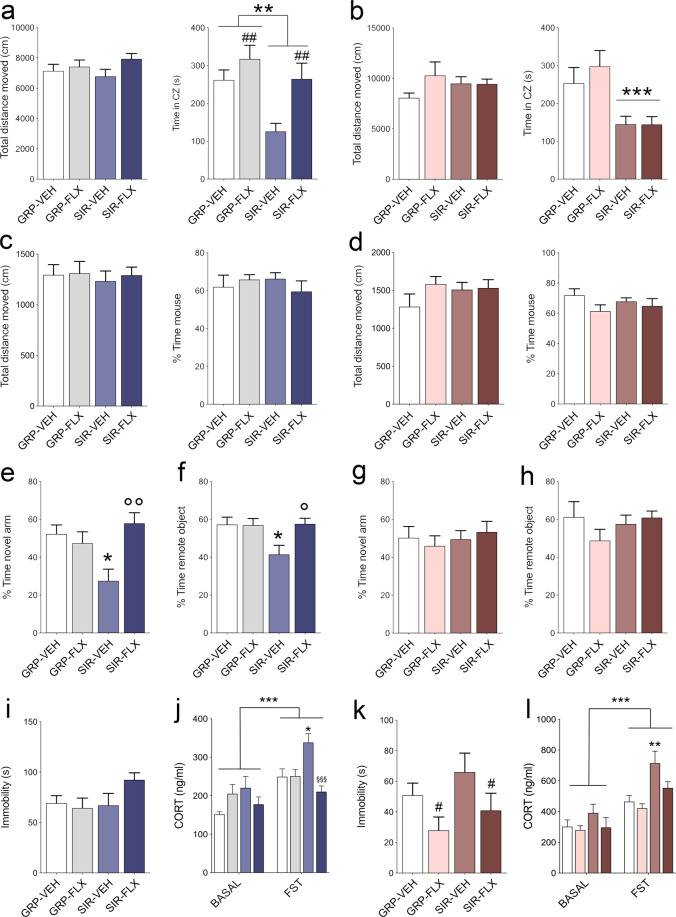
Effects of maternal SIR and FLX treatment on behavior and cognition in the offspring. **a** Total distance moved and time spent in the center zone of the open field test in male offspring. **p < 0.01, reflecting the main effect of SIR; ^##^*p* < 0.01, reflecting the main effect of FLX treatment. **b** Total distance moved and time spent in the center zone of the open field test in female offspring. ***p < 0.001, reflecting the main effect of SIR. **c** Total distance moved and percent exploration time of an unfamiliar mouse compared to a dummy object in the social interaction test in male offspring. **d** Total distance moved and percent exploration time of an unfamiliar mouse compared to a dummy object in the social interaction test in female offspring. **e** Relative exploration time (%) of the novel arm in the Y-maze test of spatial recognition memory in male offspring. °°*p* < 0.01 between SIR-VEH and SIR-FLX; *p < 0.05 between SIR-VEH and GRP-VEH. **f** Relative time (%) spent exploring the remote object in the temporal order memory test in male offspring; °*p* < 0.05 between SIR-VEH and SIR-FLX; *p < 0.05 between SIR-VEH and GRP-VEH. **g** Relative exploration time (%) of the novel arm in the Y-maze test of spatial recognition memory in female offspring. **h** Relative time (%) spent exploring the remote object in the temporal order memory test in female offspring. **i** Time spent immobile in the forced swim test (FST) test in male offspring. **j** Basal and FST-induced levels of CORT in male offspring. *p < 0.05 between SIR-VEH and GRP-VEH; ^§§§^*p* < 0.001 between SIR-FLX *vs* SIR-VEH. **k** Time spent immobile in the forced swim test (FST) test in female offspring. ^#^*p* < 0.05, reflecting the main effect of FLX treatment. **l** Basal and FST-induced levels of CORT in female offspring. **p < 0.01 between SIR-VEH and GRP-VEH; *N* = 10.11 mice per group and sex in **a**–**h**, *N* = 12-13 animals per group and sex in **i**–**l**. All values represent means ± s.e.m.

At adult age, both male and female offspring of SIR females displayed signs of increased anxiety-like behavior, as assessed in the in the open field test. Maternal SIR and FLX treatment generally decreased and increased, respectively, the time spent in the center zone in male offspring, as supported by the main effects of maternal SIR (*F*_(1,37)_ = 8.528, *p* < 0.05; Fig. 2a) and maternal FLX treatment (*F*_(1,37)_ = 9.060, *p* < 0.005; Fig. 2a). Hence, male SIR-VEH offspring displayed the strongest reduction in this measure, whereas male SIR-FLX offspring reached levels that were comparable to those measured in male GRP-VEH animals (Fig. 2a). SIR female offspring spent less time in the center zone when compared to GRP female offspring, and this effect was not influenced by maternal FLX treatment, leading to a significant main effect of maternal housing (*F*_(1,38)_ = 15.95, *p* < 0.001, Fig. 2b).

Maternal SIR and/or maternal FLX treatment did not influence sociability in the social interaction test, neither in male (Fig. 2c) nor female (Fig. 2d) offspring. Maternal SIR did, however, negatively affect cognitive performance in male (Fig. 2e, f) but not female (Fig. 2g, h) offspring, and this effect was prevented by maternal FLX treatment. In the Y-maze test of spatial recognition memory, male SIR-VEH, but not SIR-FLX, offspring displayed a marked reduction in the relative time spent in the novel arm, leading to a significant interaction between maternal housing and FLX treatment (*F*_(1,37)_ = 9.841, *p* < 0.005). Consistent with these effects, male SIR offspring displayed a deficit in the temporal order memory test, which was prevented by maternal FLX treatment (Fig. 2f). This impression was supported by the presence of a significant interaction between maternal SIR and FLX treatment (*F*_(1,37)_ = 4.571, *p* < 0.05). Both the spatial recognition memory and temporal order memory tests were not confounded by differences in general locomotor activity (Supplementary Fig. S4).

Maternal SIR and/or maternal FLX treatment did not influence the time spent immobile in the forced swim test in males (Fig. 2i), while maternal FLX treatment, however, affected this measure in females (Fig. 2k). Indeed, female offspring of FLX-treated GRP or SIR mothers displayed a reduction in the time spent immobile in the forced swim test, as supported by a significant main effect of maternal FLX treatment (*F*_(1,44)_ = 5.491, *p* < 0.05). When considering basal and stress-induced levels of CORT, we observed a main effect of testing condition in both males and females (*F*_(1,47)_ = 26.834, *p* < 0.001; *F*_(1,44)_ = 41.244, *p* < 0.001), with CORT levels increasing 30’ after the FST (Fig. 2k). Interestingly, the stress-induced elevation in CORT levels was most pronounced in SIR-VEH animals but similar between SIR-FLX offspring and VEH- or FLX-GRP offspring, suggesting that maternal FLX treatment mitigated the stress-induced hypersecretion of CORT in especially in male offspring born to SIR dams. Statistical support for these results were obtained by the main effect of FLX treatment (*F*_(1,47)_ = 6.415, *p* < 0.01) and its interaction with testing condition (*F*_(1,47)_ = 3.954, *p* < 0.05) in male animals. In females, we observed a main effect of housing (*F*_(1,44)_ = 11.298, *p* < 0.01) and treatment (*F*_(1,44)_ = 5.028, *p* < 0.05), while the interaction between treatment and testing condition just missed statistical significance.

### Maternal SIR and FLX treatment regimens affect the amygdalar transcriptome in male and female offspring

To identify possible intergenerational effects of maternal SIR and FLX treatment on brain molecular profiles, we performed next-generation mRNA sequencing (RNAseq) to compare genome-wide transcriptional changes in the amygdala of offspring born to GRP or SIR mothers with or without concomitant FLX treatment. Our primary analyses focused on contrasting transcriptional profiles between SIR-VEH and GRP-VEH adult offspring to identify possible changes induced by maternal social isolation, and between SIR-VEH and SIR-FLX offspring to explore the effects of FLX treatment under conditions of antenatal exposure to maternal SIR. Secondary analyses involving GRP-VEH and GPR-FLX groups were also conducted in order to examine the transcriptional effects of maternal FLX treatment in the absence of maternal SIR (see Supplementary Figs. S5 and S6). For all comparisons, we considered changes that passed a false discovery rate (FDR) correction set at *q* < 0.05 and suggestive associations characterized by *p* < 0.005, as performed previously in models of prenatal adversity [60]. A number of transcriptional changes identified by RNAseq were further validated using quantitative polymerase chain reaction (qPCR; Supplementary Figs. S7 and S8).

**Fig. 3:**
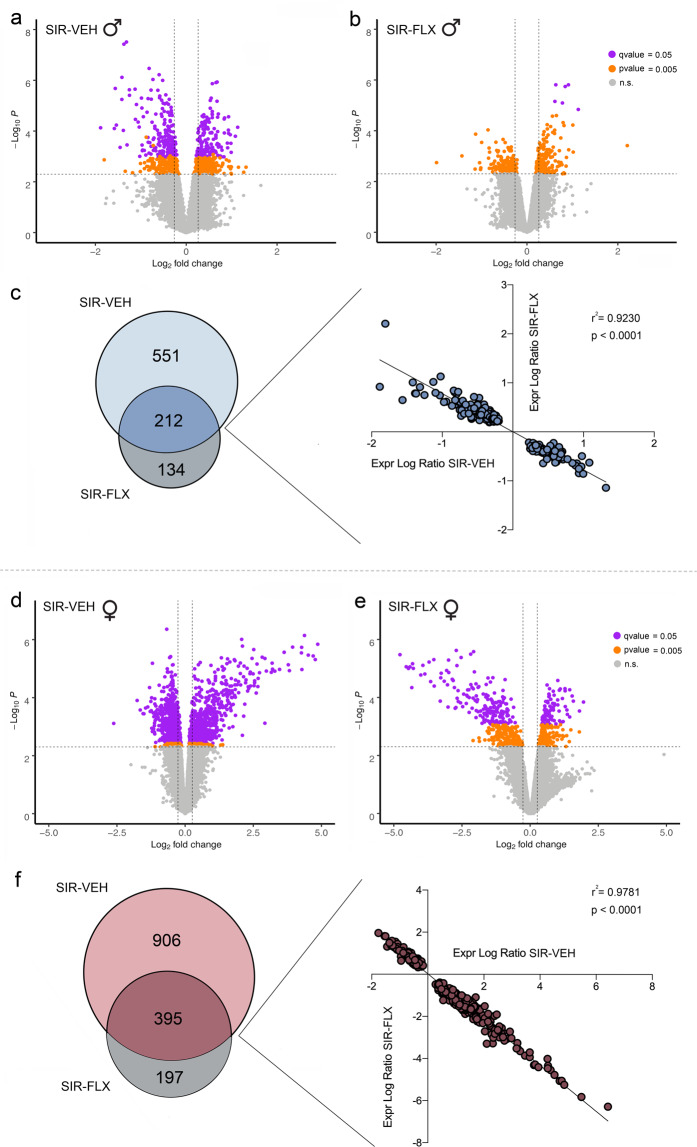
Maternal SIR and FLX treatment affect the amygdalar transcriptome in male and female offspring. Genome-wide transcriptional changes in the amygdala of offspring born to GRP or SIR mothers with or without concomitant FLX treatment. **a** Volcano plot depicting the differentially expressed genes (DEGs) in SIR-VEH *vs* GRP-VEH male offspring (FDR *q* < 0.05: purple; *p* < 0.005: orange; *n.s*: gray). **b** Volcano plot depicting the differentially expressed genes (DEGs) in SIR-FLX *vs* GRP-VEH male offspring (FDR *q* < 0.05: purple; *p* < 0.005: orange; *n.s*: gray). **c** The Venn diagram denotes the number of DEGs that are uniquely and commonly affected in SIR-VEH and SIR-FLX male offspring. The linear regression plots the expression log ratio of DEGs commonly affected in SIR-VEH and SIR-FLX offspring. **d** Volcano plot depicting the differentially expressed genes (DEGs) in SIR-VEH *vs* GRP-VEH female offspring (FDR *q* < 0.05: purple; *p* < 0.005: orange; *n.s*: gray). **e** Volcano plot depicting the differentially expressed genes (DEGs) in SIR-FLX *vs* GRP-VEH female offspring (FDR *q* < 0.05: purple; *p* < 0.005: orange; *n.s*: gray). **f** The Venn diagram denotes the number of DEGs that are uniquely and commonly affected in SIR-VEH and SIR-FLX male offspring. *N* = 5 animals per group and sex.

In male offspring, we uncovered 763 differentially expressed genes (DEGs) (346 DEGs at *q* < 0.05 and an additional 417 DEGs at *p* < 0.005; Fig. 3a) in SIR-VEH offspring compared to GRP-VEH offspring, and 346 DEGs (6 at *q* < 0.05 and an additional 340 at *p* < 0.005; Fig. 3b) in SIR-FLX offspring compared to SIR-VEH animals. Interestingly, 212 DEGs were common to both comparisons (Fig. 3c), but were deregulated with opposite direction, suggesting a counterregulatory effect of maternal FLX treatment on SIR-induced transcriptomic changes in male offspring (Fig. 3c). In females, we observed 1301 DEGs (1126 at *q* < 0.05 and additional 175 at *p* < 0.005; Fig. 3d) in SIR-VEH *versus* GRP-VEH offspring, and 592 DEGs (256 at *q* < 0.05 and additional 336 *p* < 0.005; Fig. 3e) in SIR-FLX compared to SIR-VEH animals. Once again, 395 DEGs were common, but with opposite directions, to both comparisons (Fig. 3f).

**Fig. 4:**
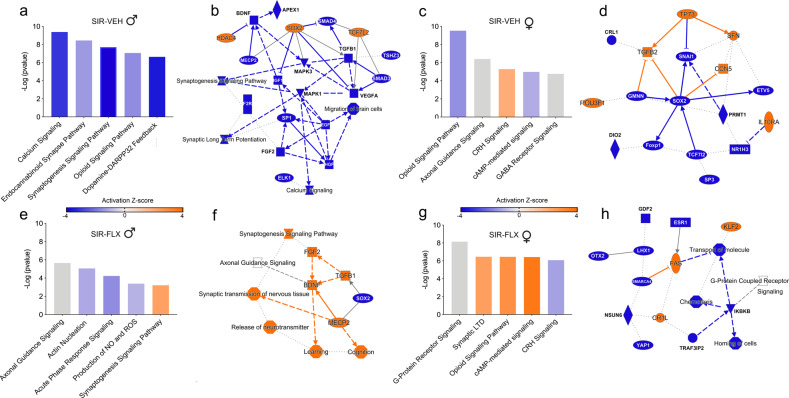
Pathway analysis of transcriptomic alterations induced by maternal SIR and FLX treatment. Ingenuity Pathway Analysis (IPA) was conducted to identify the top canonical signaling pathways affected in male and female offspring born to GRP or SIR mothers with or without concomitant FLX treatment. Orange color = positive activation Z-score (pathways) and increased expression (individual genes), blue color = negative activation Z-score (pathways) and reduced expression (individual genes). For IPA-generated overview of the main biological themes, the most significant entities (canonical pathways, upstream regulators, biological functions and molecules) are shown, with connecting lines representing specific relationships (solid lines represent a direct relationship, slashed lines an indirect relationship and dotted lines and inferred relationship). Arrows represent directionality and activation, while blunt-ended lines represent inhibition. **a** Top 5 canonical pathways annotated with DEGs in SIR-VEH *vs* GRP-VEH male offspring. **b** IPA-generated overview of the main biological themes pertaining to the comparison between SIR-VEH *vs* GRP-VEH male offspring. **c** Top 5 canonical pathways annotated with DEGs in SIR-VEH *vs* GRP-VEH female offspring. **d** IPA-generated overview of the main biological themes pertaining to the comparison between SIR-VEH *vs* GRP-VEH female offspring. **e** Top 5 canonical pathways annotated with DEGs in SIR-FLX *vs* GRP-VEH male offspring. **f** IPA-generated overview of the main biological themes pertaining to the comparison between SIR-FLX *vs* GRP-VEH male offspring. **g** Top 5 canonical pathways annotated with DEGs in SIR-FLX *vs* GRP-VEH female offspring. **h** IPA-generated overview of the main biological themes pertaining to the comparison between SIR-FLX *vs* GRP-VEH female offspring. The analyses are based on gene expression data obtained from *N* = 5 animals per group and sex.

We used Ingenuity Pathway Analysis (IPA) to identify the top canonical signaling pathways affected in male and female offspring born to GRP or SIR mothers with or without concomitant FLX treatment. In both male and female offspring, some pathways were commonly affected in the SIR-VEH and SIR-FLX groups, but with opposite activation Z-scores (Fig. 4a, c, e, g), corroborating the notion that some of the effects of SIR on transcriptional profiles were counter-regulated by FLX treatment (Fig. 3c, f). These include, for example, ‘synaptogenesis signaling’, ‘corticotropin-releasing hormone (CRH) signaling’ and ‘cyclic adenosine monophosphate (cAMP)-mediated signaling’. The corresponding DEGs annotated with each of the top 5 pathways are provided in Supplementary Tables S4 and S5 for male, and in S6 and S7 for female, offspring. A graphical summary of the major biological themes, in which the most significant entities (canonical pathways, upstream regulators and biological functions) are related to each other by IPA, is provided in provided in Fig. 4b, d for males, and in Fig. 4f, h for females.

**Fig. 5:**
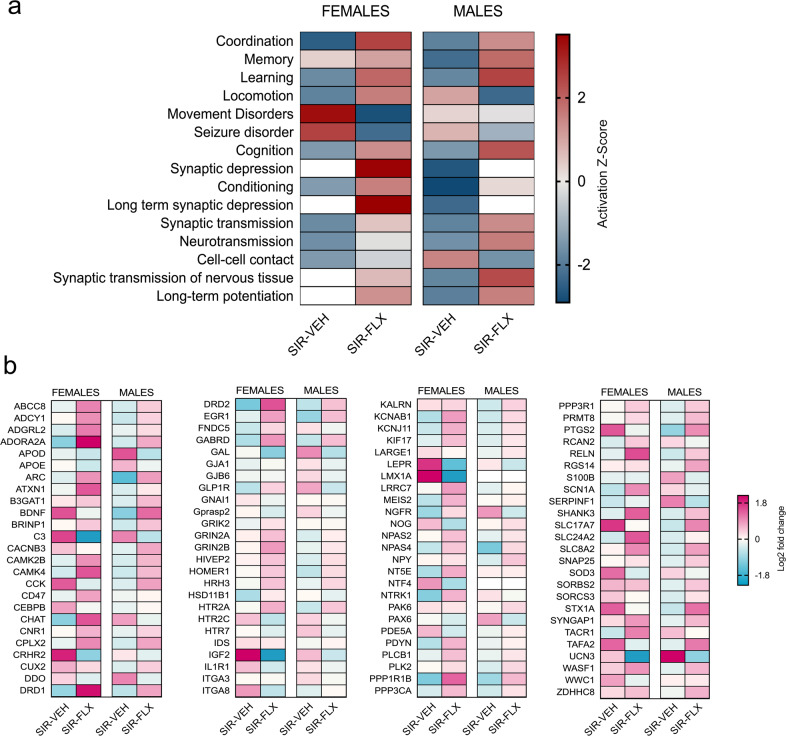
Comparison analysis of transcriptomic alterations induced by maternal SIR and FLX treatment. Ingenuity Pathway Analysis (IPA) was used to identify modules of biological functions predicted to be increased or decreased in male and female offspring born to GRP or SIR mothers with or without concomitant FLX treatment. **a** Heatmap visualization of the top 15 biological functions that are similarly or differentially affected in SIR-VEH and SIR-FLX female and male offspring. The colors of the heatmap reflect the activation Z-score of each biological function in each dataset, and ranges from dark blue (negative activation Z-score) to dark red (positive activation Z-score). **b** The gene-level heatmap graphically depicts which genes are up- or downregulated in the biological function module ‘memory’ across different datasets. The colors of the heatmap reflect the Log2 fold-change and range from blue (downregulated) to magenta (upregulated). The analyses are based on gene expression data obtained from *N* = 5 animals per group and sex.

We further analyzed how the above-mentioned gene expression changes could translate into different functional readouts, and thus possibly associate with the behavioral differences occurring in offspring born to GRP or SIR mothers with or without concomitant FLX treatment. To this aim, we performed a comparison analysis in the ‘Disease and Functions’ module of IPA. We found that the DEGs annotated with various behavioral and neuronal functions, including “learning”, “memory”, “cognition”, “synaptic transmission”, and “neurotransmission” (Fig. 5a). Consistent with the results obtained in the analysis of canonical signaling pathways (Fig. 4), the effects of SIR on transcriptional profiles were often found to be counter-regulated by FLX treatment, as indicated by opposing activation Z-scores in SIR-VEH and SIR-FLX groups (Fig. 5a). Moreover, whereas some effects in the ‘Disease and Functions’ module were similar in male and female offspring (e.g., “learning”), others differed between sex. An illustrative example of the latter is “memory”, which was found to be negatively affected only in male but not female SIR-VEH offspring (Fig. 5a). A graphical representation of the DEGs annotating with the term “memory” is provided in Fig. 5b, while the other terms are graphically depicted in Supplementary Fig. S9. Among others, these DEGs include neurotrophic factors (*Bdnf, Ntf4*), dopaminergic, glutamatergic, and serotoninergic receptors (*Drd1*, *Drd2*, *Grik2*, *Grin2a*, *Grin2b*, *Htr2a*, *Htr2c*), neuronal calmodulin kinases (*CamkIV*, *CamK2B*), and neuronal transcription factors (*Npas2*, *Npas4*). Of note, the majority of these genes were downregulated in SIR-VEH offspring, but upregulated in SIR-FLX offspring, supporting the hypothesis that maternal FLX treatment counter-regulated the effects of SIR on transcriptomic changes associated with functional terms such as “memory”.

## Discussion

Our study shows that SIR of nulliparous female mice prior to mating leads to a depression-like state that is initiated before and continued throughout pregnancy. The main behavioral outcomes observed in both SIR dams and their offspring are recapitulated in Table 1. In dams, SIR increased anxiety-like behavior in the light-dark-box test and open field test, reduced sociability in the social interaction test, increased immobility in the forced swim test, and led to blunted CORT levels in blood and excessive weight gain. These effects of SIR are in line with previous studies [49, 50] and confirm the suitability of this housing paradigm to model pre-gestational depressive-like abnormalities in female mice. We further found that maternal FLX treatment was capable of ameliorating some of the SIR-induced behavioral deficits, including social interaction deficits and increased immobility in the forced swim test, while it did not normalize SIR-induced alterations in anxiety-like behavior and CORT levels in blood. These results are consistent with previous findings [50], and with the notion that while chronic FLX treatment is effective in normalizing deficits in social behavior and the forced swim test, it is less effective on measures of anxiety [61–63]. Regardless of the pharmacological intervention, plasma CORT levels were found to be lower in SIR relative to GRP females, both at basal conditions and after exposure to the forced swim test. Moreover, the post-FST CORT levels were dissociable from the dams’ performance in the FST, such that SIR females displaying heightened immobility in the FST did not show increased plasma CORT after the test. The SIR-mediated attenuation of basal CORT release and the dissociation between the dams’ performance in the FST and post-FST CORT levels are in line with other reports suggesting a gradual decrease in basal adrenal activity in response to continuous stressors [50, 64–66], and with the emerging evidence suggesting that CORT levels may not correlate with the behavioral profile of female mice in the FST [67].

**Table 1:** Summary of the effects maternal SIR and FLX treatment on the dams and their offspring.

Test	Maternal behavior				Offspring behavior							
					Males				Females			
	Grouped		SIR		Grouped		SIR		Grouped		SIR	
	VEH	FLX	VEH	FLX	VEH	FLX	VEH	FLX	VEH	FLX	VEH	FLX
**Anxiety**												
Light-Dark box												
Latency	=	NA	↑	NA	NA	NA	NA	NA	NA	NA	NA	NA
Open-field												
% Time in Centre Zone	=	=	↓	↓	=	↑	↓	↑	=	=	↓	↓
**Social behavior**												
Social Interaction												
% Time interacting with live mouse	=	=	↓	=	=	=	=	=	=	=	=	=
**Cognitive function**												
Temporal order memory test												
% Time with remote object	NA	NA	NA	NA	=	=	↓	=	=	=	=	=
Y-maze												
% Time in the novel arm	NA	NA	NA	NA	=	=	↓	=	=	=	=	=
**FST**												
Forced swim test												
Time spent immobile	=	=	↑	=	=	=	=	=	=	↓	=	↓
**CORT**												
Basal CORT	=	=	↓	↓	=	=	=	=	=	=	=	=
Post-FST CORT	=	=	↓	↓	=	=	↑	=	=	=	↑	=

*NA* not applicable, animals not tested in these behaviors, *=* Control level and no change compared to CON-VEH, *↑* Increased measure compared to CON-VEH, *↓* Decreased measure compared to CON-VEH.

Collectively, our data suggest that SIR of nulliparous female mice prior to mating can be used as an experimental model system to study the effects of maternal depression-like states on long-term outcomes in the offspring. Importantly, this model is based on an etiologically highly relevant risk factor for depression [41–43], and allows researchers to ascertain the degree of SIR-induced abnormalities prior to conception. The latter is one of the main strengths of the model, given that the strongest risk factor for depression during pregnancy is a history of recent depression [44, 45]. Hence, unlike animal models in which aspects of prenatal depression are mimicked through selected gestational manipulations only [23, 35–40], our model presented here takes into account behavioral and physiological mal-adaptations to an etiologically relevant risk factor of depression that are initiated before and maintained throughout pregnancy. As a consequence, our approach more readily mimics the clinical situation in which depressed women become pregnant and thus present the pathology before and over the entire course of pregnancy. The presence of pre- and peri-gestational depression is usually clinically more challenging when compared to depressive-like symptoms that arise selectively during gestation, and appears to have more severe consequences for the long-term mental wellbeing of the exposed offspring [46, 47, 68]. Indeed, antenatal depression is considered a stronger risk factor for abnormal mental health trajectories in the offspring than, for example, postpartum depression [46, 69–71].

In offspring born to SIR dams, we identified a variety of behavioral and molecular abnormalities relative to offspring born to mothers that were maintained in social groups prior to conception, including increased anxiety-like behavior, cognitive impairments and alterations of the amygdalar transcriptome. Thus far, only very limited attempts have been made to investigate the effects of pre-gestational stress on exposed offspring, and currently existing studies have not incorporated social stressors [72, 73]. Moreover, prenatal stress studies often failed to include female offspring, rendering our results an important extension of prenatal depression research. In line with previous rodent studies that were based on gestational stress exposure only [33], we found that male and female offspring of SIR dams display increased anxiety-like behaviors, suggesting that this is a robust outcome of several stress-related prenatal adversities. These effects, in turn mirror reports of increased anxiety in children exposed to antenatal depression [74, 75]. Male SIR offspring were further found to display cognitive impairments in spatial recognition memory and temporal order memory. These observations parallel reports of impaired spatial learning in the Morris water maze in adult male rodents exposed to different prenatal stressors [33, 76–78], as well as clinical findings of altered cognitive development and functioning in male children exposed to maternal antenatal depression [5, 79].

Because of its effectiveness in inducing a depression-like state before and throughout pregnancy, the present model is also highly suitable to contrast possible developmental effects of chronic SSRIs under pathological (SIR females) and non-pathological (GRP females) conditions. While the majority of existing rodent studies have focused on the behavioral effects of prenatal stress during pregnancy in the absence of additional pharmacological interventions [33], or on the effects of gestational FXL treatment in otherwise non-manipulated animals [23], only a few studies have examined these factors in combination [35, 80, 81]. Moreover, only very few studies have included male and female offspring, thereby contrasting possible sex-dependent effects of antenatal depression. Here, we found that maternal FLX treatment in SIR dams prevented the development of anxiety-like behaviors in male but not female offspring. These findings are in line with previous reports demonstrating preventive effects of perinatal FLX treatment on prenatal stress-induced anxiety-like behavior in male offspring [35, 80, 81], while they are seemingly in contrast with the two existing studies that observed no effects of perinatal FLX on anxiety-like behaviors in female offpring [36, 82]. These previous studies, however, did not observe basal prenatal stress-induced anxiety-like behavior in female animals, making a direct comparison with our findings challenging. The discrepant findings reported here and before [36, 82] are likely due to the completely different nature of the antenatal stress model. Indeed, contrary to our continuous pre- and peri-gestational stress model, previous work investigating maternal FLX treatment was based on gestational restraint stress applied during the last weeks of pregnancy only [36, 82].

We further found that the cognitive deficits emerging in male (but not female) offspring of SIR mothers were prevented by maternal FLX treatment. To our knowledge, these data are the first to document preventive effects of maternal FLX treatment against the development of cognitive deficits in (male) offspring of socially isolated mothers. In a previous study in mice, Kyrianova et al. also investigated the effects of perinatal FLX on cognition in a model of prenatal stress, but observed no effects (of either the prenatal stress or FLX) in male offspring, whereas a significant effect of prenatal FLX alone on spatial memory was identified in female offspring [38, 82]. Once again, the discrepancies between our findings and those reported by Kyrianova et al. [38, 82] are likely to be accounted for by the different experimental paradigm used to induce a depressive-like state in the dams and to differences in the FLX treatment regimen. Based on the existing evidence, it appears that the restriction of antenatal stress to specific time points of gestation only yields milder cognitive outcomes in the offspring when compared to our model. Lastly, maternal SIR did not affect immobility in the FST in either male or female offspring, a finding that is not unprecedented in other models of gestational stress [33]. On the other hand, developmental FLX decreased immobility in female offspring independently of maternal housing, as seen previously [83]. Lastly, we also observed that maternal SIR led to hypersecretion of CORT in both male and female offspring 30 min after the FST. Interestingly, this effect was prevented, especially in males, by maternal FLX treatment, suggesting that antenatal depression and FLX treatment induce diametrically opposite effects on HPA axis responsivity in the offspring, as previously observed in both animal studies and human epidemiological investigations [80, 84]. Notably, the post-FST CORT levels did not correlate with the animals’ behavioral performance in the FST. Even if similar dissociation has been previously observed in female [67], and, to a lesser extent, in male [85], animals, further studies are warranted to investigate the underlying mechanisms.

Taken together, our data identify sex-specific consequences of maternal SIR and FLX treatment in the offspring. The fact that cognitive deficits only emerged in male but not female offspring of SIR mothers suggests that male offspring are more vulnerable to develop lasting cognitive deficits after prenatal exposure to maternal depression-like states than female offspring. Moreover, maternal FLX treatment in SIR dams prevented the development of anxiety-like behaviors and cognitive abnormalities in male but not female offspring, indicating that while males may be more susceptible to the detrimental effects of maternal depression-like states, they are also more responsive to preventive effects of maternal FLX treatment after SIR. The sex-specific programming of behavioral abnormalities observed here is in line with the findings from other rodent models of gestational stress exposure [86] and may be explained by a differential susceptibility of male and female placental functions to prenatal perturbations, with male placentas being more vulnerable to maternal perturbations during pregnancy than female placentas [87]. Future studies will be necessary to explore the role of the placenta in shaping sex-specific vulnerability to behavioral and cognitive abnormalities in offspring born to socially isolated mothers. Of note, while sex-specific behavioral abnormalities have also been observed in clinical studies, however, due to the high heterogeneity of available results there is currently no consensus as to whether male offspring are more vulnerable than females in response to antenatal maternal depression [79, 88].

The use of genome-wide RNA-sequencing further showed that the behavioral abnormalities identified in offspring born to SIR mothers were accompanied by widespread transcriptomic changes in the amygdala, a brain region known to be critical for emotional processing and affective behaviors [58, 59]. Genome-wide transcriptional signatures of gestational stress and SSRI treatment in adult animals have only been investigated in one previous study, which observed no significantly altered transcripts in the hippocampus or hypothalamus of male rat offspring [89]. In contrast, our data demonstrate that maternal SIR leads to changes in the transcriptional profile of the offspring’s amygdala, some of which were reversed by maternal treatment with FLX. It thus appears that that the restriction of antenatal stress to a specific time-window of gestation only may induce milder effects on the offspring’s brain transcriptome as compared to continuous pre- and perinatal stress exposure. Overall, the DEGs identified in our study were annotated with canonical pathways important for nervous system development and neurotransmitter function, such as ‘synaptogenesis signaling’, ‘axonal guidance signaling’ and ‘opioid signaling pathway’. Consistent with the behavioral and cognitive effects, some of the transcriptomic changes induced by maternal SIR were found to be sex-dependent. Synaptogenesis signaling, for example, was exclusively affected in male offspring, whereas CRH signaling was altered in females only. Sex-dependent effects were also evident when considering how the identified DEGs annotated with specific behavioral functions. The gene modules annotating with the ‘Disease and Functions’ terms “learning”, “cognition” and “memory” are illustrative examples of the sex-specific effects, with “memory” presenting a negative activation score only in male offspring (Fig. 5a). This male-specific effect parallels, and is consistent with, the preferential vulnerability of male offspring to develop spatial recognition and temporal order memory deficits after prenatal exposure to SIR that are rescued by maternal treatment with FLX. Similar sex-specific vulnerability was also evident in the analyses of other gene modules, such as ‘synaptic depression’, ‘long term potentiation’ and ‘synaptic transmission of nervous tissue’, which might also contribute to the sex-dependent memory impairments. When exploring the specific DEGs that annotate with “memory”, we uncovered multiple candidates previously singularly associated with prenatal stress and SSRI treatment, such as neurotrophic factors [35, 36, 49, 90, 91], *Reelin* [92–94], and serotonin receptors [73, 95, 96]. Specifically, we observed reduced and increased amygdalar *Bdnf* expression in male and female SIR offspring, respectively, corroborating the sex-dependent effects of prenatal stress on this neurotrophic factor [35, 36, 90, 91, 97, 98]. Moreover, we found serotonin receptor expression to be dysregulated in male, and to a lesser extent in female, offspring, extending previous findings that suggest long-lasting effects of prenatal stress on serotoninergic functioning at different time point during development, which, in our hands, are partially normalized by perinatal FLX [73, 95, 96]. Interestingly, female SIR offspring displayed quantitively more transcriptomic changes in the amygdala than male SIR offspring, despite the fact that they showed fewer behavioral changes when compared to males. While some of these changes may represent sex-specific allostatic adaptations to maternal SIR, they may also point towards amygdala-related structural and functional brain abnormalities not investigated in the present study.

In line with the partial prevention of SIR-induced behavioral and cognitive effects by maternal FLX treatment, the pharmacological intervention also counter-regulated some of the transcriptomic consequences of maternal SIR. Examples for such effects include DEGs annotated with the ‘Disease and Functions’ modules “learning”, “cognition” “synaptic transmission” and, in males only, “memory”, DEGs annotating with synaptogenesis and CRH signaling in males and females, respectively, as well as BDNF and serotonin receptors. These data provide molecular evidence for the hypothesis that maternal FLX treatment is capable of attenuating the lasting transcriptomic consequences in a model of prenatal depression-like states. Moreover, they provide a molecular mechanism through which FLX could counteract the negative effects of maternal SIR on cognition in male offspring. Collectively, our transcriptomic data offer an unbiased picture of the long-term molecular alterations induced by maternal depression in adult offspring, and highlights specific pathways that are convergent targets of both maternal pathology and treatment. Further studies will be necessary to uncover the precise relationship between these gene expression changes and the specific behavioral alterations brought about by maternal SIR and FLX treatment.

Our study is associated with a number of limitations. First, we did not assess the possible impact of SIR on postpartum maternal behavior and its association with behavioral development in the offspring. Thus, it remains unknown if maternal SIR negatively affects maternal-pup interactions and, if so, whether maternal FLX treatment would influence these relationships. Previous work reports that perinatal FLX reverses the lasting effects of pre-gestational stress on various physiological parameters in the dam, but not on maternal behavior [72], suggesting that the beneficial effect of FLX on the offspring of SIR animals could be mediated by other, in utero, mechanisms. Dissecting the relative contribution of pre- and postnatal factors using neonatal cross-fostering designs will help addressing this issue in future studies. Second, our study was not designed to assess causal relationships between the transcriptomic and behavioral changes in the present model. As a consequence, the relationship between sex-dependent behavioral alterations and transcriptomic profiles remains descriptive. Despite these limitations, we conclude that the mouse model presented here is a valuable experimental system to study the effects of maternal depression and perinatal SSRI treatment on brain and behavioral development offspring. Key strengths of this model include (i) its etiologically relevant basis and ontopathogenic validity, (ii) its experimental timeline, allowing the ascertainment of pre-gestational depressive-like behavior, (iii) and its efficacy in recapitulating many aspects of prenatal depression. The future use of this model may thus help advance our understanding of the impact of maternal depression with or without SSRI treatment on offspring wellbeing.

## Supplementary information

Supplement 1

## References

[1] Graignic-Philippe R, Dayan J, Chokron S, Jacquet AY, Tordjman S (2014). Effects of prenatal stress on fetal and child development: a critical literature review. Neurosci Biobehav Rev.

[2] Fisher J, Cabral de Mello M, Patel V, Rahman A, Tran T, Holton S (2012). Prevalence and determinants of common perinatal mental disorders in women in low- and lower-middle-income countries: a systematic review. Bull World Health Organ.

[3] Melville JL, Gavin A, Guo Y, Fan MY, Katon WJ (2010). Depressive disorders during pregnancy: prevalence and risk factors in a large urban sample. Obstet Gynecol.

[4] Rogers A, Obst S, Teague SJ, Rossen L, Spry EA, Macdonald JA et al. Association between maternal perinatal depression and anxiety and child and adolescent development: a meta-analysis. JAMA Pediatr. 2020; 10.1001/jamapediatrics.2020.2910. 10.1001/jamapediatrics.2020.2910 PMC7490743 32926075

[5] Loomans EM, van Dijk AE, Vrijkotte TG, van Eijsden M, Stronks K, Gemke RJ (2013). Psychosocial stress during pregnancy is related to adverse birth outcomes: results from a large multi-ethnic community-based birth cohort. Eur J Public Health.

[6] Huybrechts KF, Palmsten K, Mogun H, Kowal M, Avorn J, Setoguchi-Iwata S (2013). National trends in antidepressant medication treatment among publicly insured pregnant women. Gen Hosp Psychiatry.

[7] Xing D, Wu R, Chen L, Wang T (2020). Maternal use of antidepressants during pregnancy and risks for adverse perinatal outcomes: a meta-analysis. J Psychosom Res.

[8] Mitchell AA, Gilboa SM, Werler MM, Kelley KE, Louik C, Hernandez-Diaz S (2011). Medication use during pregnancy, with particular focus on prescription drugs: 1976-2008. Am J Obstet Gynecol.

[9] Oberlander TF, Gingrich JA, Ansorge MS (2009). Sustained neurobehavioral effects of exposure to SSRI antidepressants during development: molecular to clinical evidence. Clin Pharm Ther.

[10] Hiemke C, Hartter S (2000). Pharmacokinetics of selective serotonin reuptake inhibitors. Pharm Ther.

[11] Heikkinen T, Ekblad U, Kero P, Ekblad S, Laine K (2002). Citalopram in pregnancy and lactation. Clin Pharm Ther.

[12] Heikkinen T, Ekblad U, Palo P, Laine K (2003). Pharmacokinetics of fluoxetine and norfluoxetine in pregnancy and lactation. Clin Pharm Ther.

[13] Vitalis T, Parnavelas JG (2003). The role of serotonin in early cortical development. Dev Neurosci.

[14] Whitaker-Azmitia PM (2001). Serotonin and brain development: role in human developmental diseases. Brain Res Bull.

[15] Reis M, Kallen B (2010). Delivery outcome after maternal use of antidepressant drugs in pregnancy: an update using Swedish data. Psychol Med.

[16] Chang Q, Ma XY, Xu XR, Su H, Wu QJ, Zhao YH (2020). Antidepressant use in depressed women during pregnancy and the risk of preterm birth: a systematic review and meta-analysis of 23 cohort studies. Front Pharm.

[17] Kallen B, Olausson PO (2008). Maternal use of selective serotonin re-uptake inhibitors and persistent pulmonary hypertension of the newborn. Pharmacoepidemiol Drug Saf.

[18] Boukhris T, Berard A (2015). Selective serotonin reuptake inhibitor use during pregnancy and the risk of autism spectrum disorders: a review. J Pediatr Genet.

[19] Boukhris T, Sheehy O, Berard A (2017). Antidepressant use in pregnancy and the risk of attention deficit with or without hyperactivity disorder in children. Paediatr Perinat Epidemiol.

[20] Croen LA, Grether JK, Yoshida CK, Odouli R, Hendrick V (2011). Antidepressant use during pregnancy and childhood autism spectrum disorders. Arch Gen Psychiatry.

[21] Laine K, Heikkinen T, Ekblad U, Kero P (2003). Effects of exposure to selective serotonin reuptake inhibitors during pregnancy on serotonergic symptoms in newborns and cord blood monoamine and prolactin concentrations. Arch Gen Psychiatry.

[22] Oberlander TF, Grunau R, Mayes L, Riggs W, Rurak D, Papsdorf M (2008). Hypothalamic-pituitary-adrenal (HPA) axis function in 3-month old infants with prenatal selective serotonin reuptake inhibitor (SSRI) antidepressant exposure. Early Hum Dev.

[23] Millard SJ, Weston-Green K, Newell KA (2017). The effects of maternal antidepressant use on offspring behaviour and brain development: Implications for risk of neurodevelopmental disorders. Neurosci Biobehav Rev.

[24] Biffi A, Cantarutti A, Rea F, Locatelli A, Zanini R, Corrao G (2020). Use of antidepressants during pregnancy and neonatal outcomes: an umbrella review of meta-analyses of observational studies. J Psychiatr Res.

[25] Petersen I, Evans S, Nazareth I (2014). Prenatal exposure to selective serotonin reuptake inhibitors and autistic symptoms in young children: another red herring?. Br J Psychiatry.

[26] Ashman SB, Dawson G, Panagiotides H, Yamada E, Wilkinson CW (2002). Stress hormone levels of children of depressed mothers. Dev Psychopathol.

[27] Davalos DB, Yadon CA, Tregellas HC (2012). Untreated prenatal maternal depression and the potential risks to offspring: a review. Arch Women’s Ment Health.

[28] Glover V (2014). Maternal depression, anxiety and stress during pregnancy and child outcome; what needs to be done. Best Pr Res Clin Obstet Gynaecol.

[29] Hammen C, Brennan PA (2003). Severity, chronicity, and timing of maternal depression and risk for adolescent offspring diagnoses in a community sample. Arch Gen Psychiatry.

[30] Gentile S (2017). Untreated depression during pregnancy: Short- and long-term effects in offspring. A systematic review. Neuroscience.

[31] Nulman I, Koren G, Rovet J, Barrera M, Pulver A, Streiner D (2012). Neurodevelopment of children following prenatal exposure to venlafaxine, selective serotonin reuptake inhibitors, or untreated maternal depression. Am J Psychiatry.

[32] Yonkers KA, Wisner KL, Stewart DE, Oberlander TF, Dell DL, Stotland N (2009). The management of depression during pregnancy: a report from the American Psychiatric Association and the American College of Obstetricians and Gynecologists. Gen Hosp Psychiatry.

[33] Weinstock M (2017). Prenatal stressors in rodents: effects on behavior. Neurobiol Stress.

[34] Glover ME, Clinton SM (2016). Of rodents and humans: a comparative review of the neurobehavioral effects of early life SSRI exposure in preclinical and clinical research. Int J Dev Neurosci.

[35] Boulle F, Pawluski JL, Homberg JR, Machiels B, Kroeze Y, Kumar N (2016). Prenatal stress and early-life exposure to fluoxetine have enduring effects on anxiety and hippocampal BDNF gene expression in adult male offspring. Dev Psychobiol.

[36] Boulle F, Pawluski JL, Homberg JR, Machiels B, Kroeze Y, Kumar N (2016). Developmental fluoxetine exposure increases behavioral despair and alters epigenetic regulation of the hippocampal BDNF gene in adult female offspring. Horm Behav.

[37] Glover ME, Pugh PC, Jackson NL, Cohen JL, Fant AD, Akil H (2015). Early-life exposure to the SSRI paroxetine exacerbates depression-like behavior in anxiety/depression-prone rats. Neuroscience.

[38] Kiryanova V, Meunier SJ, Vecchiarelli HA, Hill MN, Dyck RH (2016). Effects of maternal stress and perinatal fluoxetine exposure on behavioral outcomes of adult male offspring. Neuroscience.

[39] Kiryanova V, Smith VM, Dyck RH, Antle MC (2017). Circadian behavior of adult mice exposed to stress and fluoxetine during development. Psychopharmacol (Berl).

[40] Rayen I, van den Hove DL, Prickaerts J, Steinbusch HW, Pawluski JL (2011). Fluoxetine during development reverses the effects of prenatal stress on depressive-like behavior and hippocampal neurogenesis in adolescence. PLoS One.

[41] Bekhet AK, Zauszniewski JA, Nakhla WE (2008). Loneliness: a concept analysis. Nurs Forum.

[42] Blazer DG, Hybels CF (2005). Origins of depression in later life. Psychol Med.

[43] Heinrich LM, Gullone E (2006). The clinical significance of loneliness: a literature review. Clin Psychol Rev.

[44] Dietz PM, Williams SB, Callaghan WM, Bachman DJ, Whitlock EP, Hornbrook MC (2007). Clinically identified maternal depression before, during, and after pregnancies ending in live births. Am J Psychiatry.

[45] Stewart DE (2011). Clinical practice. Depression during pregnancy. N. Engl J Med.

[46] Pawlby S, Hay DF, Sharp D, Waters CS, O’Keane V (2009). Antenatal depression predicts depression in adolescent offspring: prospective longitudinal community-based study. J Affect Disord.

[47] Plant DT, Pawlby S, Sharp D, Zunszain PA, Pariante CM (2016). Prenatal maternal depression is associated with offspring inflammation at 25 years: a prospective longitudinal cohort study. Transl Psychiatry.

[48] Koike H, Ibi D, Mizoguchi H, Nagai T, Nitta A, Takuma K (2009). Behavioral abnormality and pharmacologic response in social isolation-reared mice. Behav Brain Res.

[49] Kumari A, Singh P, Baghel MS, Thakur MK (2016). Social isolation mediated anxiety like behavior is associated with enhanced expression and regulation of BDNF in the female mouse brain. Physiol Behav.

[50] Martin AL, Brown RE (2010). The lonely mouse: verification of a separation-induced model of depression in female mice. Behav Brain Res.

[51] Huang Q, Zhou Y, Liu LY (2017). Effect of post-weaning isolation on anxiety- and depressive-like behaviors of C57BL/6J mice. Exp Brain Res.

[52] Lin S, Li X, Chen YH, Gao F, Chen H, Hu NY et al. Social isolation during adolescence induces anxiety behaviors and enhances firing activity in BLA pyramidal neurons via mGluR5 upregulation. Mol Neurobiol. 2017; 10.1007/s12035-017-0766-1. 10.1007/s12035-017-0766-1 28914419

[53] O’Keefe LM, Doran SJ, Mwilambwe-Tshilobo L, Conti LH, Venna VR, McCullough LD (2014). Social isolation after stroke leads to depressive-like behavior and decreased BDNF levels in mice. Behav Brain Res.

[54] Ueno H, Suemitsu S, Murakami S, Kitamura N, Wani K, Okamoto M (2017). Region-specific impairments in parvalbumin interneurons in social isolation-reared mice. Neuroscience.

[55] Mumtaz F, Khan MI, Zubair M, Dehpour AR (2018). Neurobiology and consequences of social isolation stress in animal model-A comprehensive review. Biomed Pharmacother.

[56] Meyer U, Spoerri E, Yee BK, Schwarz MJ, Feldon J (2010). Evaluating early preventive antipsychotic and antidepressant drug treatment in an infection-based neurodevelopmental mouse model of schizophrenia. Schizophr Bull.

[57] Mueller FS, Scarborough J, Schalbetter SM, Richetto J, Kim E, Couch A, et al. Behavioral, neuroanatomical, and molecular correlates of resilience and susceptibility to maternal immune activation. Mol Psychiatry. 2020, 10.1038/s41380-020-00952-8. 10.1038/s41380-020-00952-8 PMC7850974 33230204

[58] Dolan RJ (2002). Emotion, cognition, and behavior. Science.

[59] Phelps EA, LeDoux JE (2005). Contributions of the amygdala to emotion processing: from animal models to human behavior. Neuron.

[60] Page NF, Gandal M, Estes M, Cameron S, Buth J, Parhami S, et al. Alterations in retrotransposition, synaptic connectivity, and myelination implicated by transcriptomic changes following maternal immune activation in non-human primates. Biol. Psychiatry. 2021, 10.1016/j.biopsych.2020.10.016. 10.1016/j.biopsych.2020.10.016 PMC8052273 33386132

[61] Gray VC, Hughes RN (2015). Drug-, dose- and sex-dependent effects of chronic fluoxetine, reboxetine and venlafaxine on open-field behavior and spatial memory in rats. Behav Brain Res.

[62] Mutlu O, Gumuslu E, Ulak G, Celikyurt IK, Kokturk S, Kir HM (2012). Effects of fluoxetine, tianeptine and olanzapine on unpredictable chronic mild stress-induced depression-like behavior in mice. Life Sci.

[63] Sanchez C, Meier E (1997). Behavioral profiles of SSRIs in animal models of depression, anxiety and aggression. Are they all alike?. Psychopharmacol (Berl).

[64] Beitia G, Garmendia L, Azpiroz A, Vegas O, Brain PF, Arregi A (2005). Time-dependent behavioral, neurochemical, and immune consequences of repeated experiences of social defeat stress in male mice and the ameliorative effects of fluoxetine. Brain Behav Immun.

[65] Konkle AT, Baker SL, Kentner AC, Barbagallo LS, Merali Z, Bielajew C (2003). Evaluation of the effects of chronic mild stressors on hedonic and physiological responses: sex and strain compared. Brain Res.

[66] Nichols DJ, Chevins PF (1981). Effects of housing on corticosterone rhythm and stress responses in female mice. Physiol Behav.

[67] Kokras N, Krokida S, Varoudaki TZ, Dalla C. Do corticosterone levels predict female depressive-like behavior in rodents? J Neurosci Res. 2020, 10.1002/jnr.24686. 10.1002/jnr.24686 32640495

[68] Plant DT, Pariante CM, Sharp D, Pawlby S (2015). Maternal depression during pregnancy and offspring depression in adulthood: role of child maltreatment. Br J Psychiatry.

[69] Davis EP, Glynn LM, Schetter CD, Hobel C, Chicz-Demet A, Sandman CA (2007). Prenatal exposure to maternal depression and cortisol influences infant temperament. J Am Acad Child Adolesc Psychiatry.

[70] Pearson RM, Evans J, Kounali D, Lewis G, Heron J, Ramchandani PG (2013). Maternal depression during pregnancy and the postnatal period: risks and possible mechanisms for offspring depression at age 18 years. JAMA Psychiatry.

[71] Van Batenburg-Eddes T, Brion MJ, Henrichs J, Jaddoe VW, Hofman A, Verhulst FC (2013). Parental depressive and anxiety symptoms during pregnancy and attention problems in children: a cross-cohort consistency study. J Child Psychol Psychiatry.

[72] Gemmel M, Harmeyer D, Bogi E, Fillet M, Hill LA, Hammond GL (2018). Perinatal fluoxetine increases hippocampal neurogenesis and reverses the lasting effects of pre-gestational stress on serum corticosterone, but not on maternal behavior, in the rat dam. Behav Brain Res.

[73] Gemmel M, Kokras N, Dalla C, Pawluski JL (2018). Perinatal fluoxetine prevents the effect of pre-gestational maternal stress on 5-HT in the PFC, but maternal stress has enduring effects on mPFC synaptic structure in offspring. Neuropharmacology.

[74] Davis EP, Sandman CA (2012). Prenatal psychobiological predictors of anxiety risk in preadolescent children. Psychoneuroendocrinology.

[75] Gerardin P, Wendland J, Bodeau N, Galin A, Bialobos S, Tordjman S (2011). Depression during pregnancy: is the developmental impact earlier in boys? A prospective case-control study. J Clin Psychiatry.

[76] Ratajczak P, Kus K, Murawiecka P, Slodzinska I, Giermaziak W, Nowakowska E (2015). Biochemical and cognitive impairments observed in animal models of schizophrenia induced by prenatal stress paradigm or methylazoxymethanol acetate administration. Acta Neurobiol Exp (Wars).

[77] Weinstock M (2008). The long-term behavioural consequences of prenatal stress. Neurosci Biobehav Rev.

[78] Barzegar M, Sajjadi FS, Talaei SA, Hamidi G, Salami M (2015). Prenatal exposure to noise stress: anxiety, impaired spatial memory, and deteriorated hippocampal plasticity in postnatal life. Hippocampus.

[79] Van den Bergh BRH, van den Heuvel MI, Lahti M, Braeken M, de Rooij SR, Entringer S, et al. Prenatal developmental origins of behavior and mental health: the influence of maternal stress in pregnancy. Neurosci Biobehav Rev. 2017, 10.1016/j.neubiorev.2017.07.003. 10.1016/j.neubiorev.2017.07.003 28757456

[80] Salari AA, Fatehi-Gharehlar L, Motayagheni N, Homberg JR (2016). Fluoxetine normalizes the effects of prenatal maternal stress on depression- and anxiety-like behaviors in mouse dams and male offspring. Behav Brain Res.

[81] Mikhailenko VA, Butkevich IP (2019). Prenatal stimulation of 5-HT1A receptors improves adaptive behavior in prenatally stressed rats. Bull Exp Biol Med.

[82] Kiryanova V, Meunier SJ, Dyck RH (2017). Behavioural outcomes of adult female offspring following maternal stress and perinatal fluoxetine exposure. Behav Brain Res.

[83] Gobinath AR, Workman JL, Chow C, Lieblich SE, Galea LA (2016). Maternal postpartum corticosterone and fluoxetine differentially affect adult male and female offspring on anxiety-like behavior, stress reactivity, and hippocampal neurogenesis. Neuropharmacology.

[84] Osborne S, Biaggi A, Chua TE, Du Preez A, Hazelgrove K, Nikkheslat N (2018). Antenatal depression programs cortisol stress reactivity in offspring through increased maternal inflammation and cortisol in pregnancy: The Psychiatry Research and Motherhood - Depression (PRAM-D) Study. Psychoneuroendocrinology.

[85] Abel EL (1994). Behavioral and physiological effects of different water depths in the forced swim test. Physiol Behav.

[86] Mueller BR, Bale TL (2008). Sex-specific programming of offspring emotionality after stress early in pregnancy. J Neurosci.

[87] Bale TL (2016). The placenta and neurodevelopment: sex differences in prenatal vulnerability. Dialogues Clin Neurosci.

[88] Stein A, Pearson RM, Goodman SH, Rapa E, Rahman A, McCallum M (2014). Effects of perinatal mental disorders on the fetus and child. Lancet.

[89] Bourke CH, Stowe ZN, Neigh GN, Olson DE, Owens MJ (2013). Prenatal exposure to escitalopram and/or stress in rats produces limited effects on endocrine, behavioral, or gene expression measures in adult male rats. Neurotoxicol Teratol.

[90] Boersma GJ, Lee RS, Cordner ZA, Ewald ER, Purcell RH, Moghadam AA (2014). Prenatal stress decreases Bdnf expression and increases methylation of Bdnf exon IV in rats. Epigenetics.

[91] Zheng Y, Fan W, Zhang X, Dong E (2016). Gestational stress induces depressive-like and anxiety-like phenotypes through epigenetic regulation of BDNF expression in offspring hippocampus. Epigenetics.

[92] Dong E, Dzitoyeva SG, Matrisciano F, Tueting P, Grayson DR, Guidotti A (2015). Brain-derived neurotrophic factor epigenetic modifications associated with schizophrenia-like phenotype induced by prenatal stress in mice. Biol Psychiatry.

[93] Dong E, Tueting P, Matrisciano F, Grayson DR, Guidotti A (2016). Behavioral and molecular neuroepigenetic alterations in prenatally stressed mice: relevance for the study of chromatin remodeling properties of antipsychotic drugs. Transl Psychiatry.

[94] Palacios-Garcia I, Lara-Vasquez A, Montiel JF, Diaz-Veliz GF, Sepulveda H, Utreras E (2015). Prenatal stress down-regulates Reelin expression by methylation of its promoter and induces adult behavioral impairments in rats. PLoS One.

[95] Sowa J, Hess G (2020). Prenatal stress-related alterations in synaptic transmission and 5-HT7 receptor-mediated effects in the rat dorsal raphe nucleus are ameliorated by the 5-HT7 receptor antagonist SB 269970. Eur J Neurosci.

[96] Akatsu S, Ishikawa C, Takemura K, Ohtani A, Shiga T (2015). Effects of prenatal stress and neonatal handling on anxiety, spatial learning and serotonergic system of male offspring mice. Neurosci Res.

[97] St-Cyr S, McGowan PO (2015). Programming of stress-related behavior and epigenetic neural gene regulation in mice offspring through maternal exposure to predator odor. Front Behav Neurosci.

[98] Panetta P, Berry A, Bellisario V, Capoccia S, Raggi C, Luoni A (2017). Long-Term Sex-Dependent Vulnerability to Metabolic challenges in Prenatally Stressed Rats. Front Behav Neurosci.

