# Supplementary material for: A novel murine model to study the impact of maternal depression and antidepressant treatment on biobehavioral functions in the offspring
Source: Mol Psychiatry. 2021 May 17;26(11):6756–72. doi: 10.1038/s41380-021-01145-7 (PMC8760069; doi:10.1038/s41380-021-01145-7)
Supplement: Supplementary file 1 — Supplement 1 [file 41380_2021_1145_MOESM1_ESM.docx]

**Supplementary Information**

**­A novel murine model to study the impact of maternal depression and antidepressant treatment on biobehavioral functions in the offspring**

Joseph Scarborough^1^, Flavia S. Mueller^1^, Ulrike Weber-Stadlbauer^1,2^, Daniele Mattei^1,3^, Lennart Opitz^4^, Annamaria Cattaneo^5,6^, Juliet Richetto^1,2^*

^1^Institute of Pharmacology and Toxicology, University of Zurich-Vetsuisse, Zurich, Switzerland.

^2^Neuroscience Center Zurich, University of Zurich and ETH Zurich, Zurich, Switzerland.

^3^Department of Neurology, Icahn School of Medicine at Mount Sinai Hospital, New York, United States

^4^Functional Genomics Center Zurich, Swiss Federal Institute of Technology and University of Zurich, 8057 Zurich, Switzerland.

^5^Biological Psychiatry Unit, IRCCS Fatebenefratelli San Giovanni di Dio, via Pilastroni 4, Brescia, Italy

^6^Department of Pharmacological and Biomolecular Sciences, University of Milan, via Balzaretti 9 - 20133 - Milan, Italy

***Correspondence:**

Dr. Juliet Richetto, Ph.D., Institute of Pharmacology and Toxicology, University of Zurich-Vetsuisse, Winterthurerstrasse 260, 8057 Zurich, Switzerland.

E-mail: [juliet.richetto@uzh.ch](mailto:juliet.richetto@uzh.ch); Tel.: +41 44 635 87 77; Fax.: +41 44 635 89 10.

**Supplementary Materials and Methods**

***Animals***

All procedures described in the present study had been previously approved by the Cantonal Veterinarian’s Office of Zurich, and all efforts were made to minimize the number of animals used and their suffering. C57BL6/N mice were housed in individually ventilated cages (IVCs) in a specific-pathogen-free (SPF) holding room, which was temperature- and humidity-controlled (21 ± 3 °C, 50 ± 10%), and kept under a reversed light–dark cycle (lights off: 09:00 AM–09.00 PM). All animals had ad libitum access to the same food (Kliba 3436, Kaiseraugst, Switzerland) and water throughout the entire study.

***Allocation, treatment, and mating of the dams***

40 group-housed females and 40 socially isolated females were included in the study (**Supplementary Table S1)**. On postnatal day (PND) 21, female mice were randomly selected and allocated to group housing of 4 or to social isolation for 7 weeks. After 7 weeks of social isolation rearing (SIR) or group housing (GRP), SIR and GRP animals were randomly divided into 2 subgroups (comprising 20 animals each) that were treated either with fluoxetine (FLX; 10mg/kg – dissolved in drinking water) or with vehicle (drinking water). The animals were kept in this housing regimen for 3 more weeks, after which they were subjected to behavioral testing (see below) and mating. FLX treatment continued throughout testing, mating and up until weaning of the offspring. The consumption of water or drug solution was monitored daily to allow for the continuous titration of the drug solution in order to maintain a correct dosing of 10mg/kg. At every titration, the pre-existing drug solution was replaced with fresh solution. The dose and administration mode were established and validated before^1^.

Timed-pregnant animals were generated via on-site breeding, which began after the 10 weeks of SIR or GRP (see above). To this end, 40 female animals deriving from the above-mentioned subgroups were subjected to a timed-mating procedure as previously described^2,3^. Successful mating was verified by the presence of a vaginal plug, upon which dams that had previously been socially isolated were housed individually throughout gestation. Dams that had previously been group housed were housed in groups of two up until 2 days prior to delivery of the pups in order to keep each litter as a single unit. The animals were left undisturbed until the offspring reached PND 14. At this point, dams were treated with FLX through a previously validated micropipette-guided drug administration (MDA) procedure^4^ in order to exclude the possibility that the pups could directly access FLX through the drinking water, and assured that the offspring were exposed to FLX exclusively via breastfeeding for the whole duration of the weaning period.

| **Maternal Housing** | **Animals** | **Treatment** | **Testing*** | **Mating** | **Final litters** |
| --- | --- | --- | --- | --- | --- |
| SIR | 40F | 20 VEH | 10 (SI, OF) | 10 | 9 |
|  |  |  | 10 (SI, OF, FST, CORT) | - |  |
|  |  | 20 FLX | 10 (SI, OF) | 10 | 9 |
|  |  |  | 10 (SI, OF, FST, CORT) | - |  |
| GRP | 40F | 20 VEH | 10 (SI, OF) | 10 | 8 |
|  |  |  | 10 (SI, OF, FST, CORT) | - |  |
|  |  | 20 FLX | 10 (SI, OF) | 10 | 7 |
|  |  |  | 10 (SI, OF, FST, CORT) | - |  |

**Supplementary Table S1.** Number and allocation of socially isolated (SIR) and group-housed (GRP) female animals. The table also specifies the final number of litters in each group. FLX, fluoxetine; VEH, vehicle. *see below for information regarding testing. The number of dams and litters included in each group was based on previous studies conducted in our laboratory based on other prenatal manipulation models.

***Allocation and testing of the offspring***

Offspring of socially-isolated or group-housed mothers that were treated with VEH or FLX were weaned on PND 21. Littermates of the same sex were caged separately and maintained in groups of 3 to 5 animals per cage. They were kept in the same vivarium and caging systems (IVCs) and were maintained under a reversed light–dark cycle and *ad libitum* food access as described above.

The offspring were left undisturbed until PND 70, after which both female and male animals were subjected to a battery of behavioral tests comprising: a light-dark box test, an open field test, a social interaction test, a Y-maze spatial recognition test and a temporal order memory test. A separate cohort of animals was subjected to basal corticosterone (CORT) measurements (see below), a forced swim test (FST) and CORT sampling 30’ after the FST. The same testing order was always applied to each animal, with 3 to 4 testing-free resting days being imposed between each test. Only 1-2 offspring per sex in each litter were randomly assigned to behavioral testing in order to minimize potential confounds arising from litter effects^5,6^. The number of offspring used for behavioral testing in each experimental condition is provided in the corresponding figure legends (see *Figure 2* in the main text) and in **Supplementary Table S2**.

| **Maternal Housing** | **Maternal Treatment** | **Resulting group** | **Animals** | **Extra FST cohort** |
| --- | --- | --- | --- | --- |
| SIR | VEH | SIR-VEH | 10 m | 13 m |
|  |  |  | 11 f | 12 f |
|  | FLX | SIR-FLX | 10 m | 13 m |
|  |  |  | 10 f | 12 f |
| Group-housing | VEH | GRP-VEH | 11 m | 12 m |
|  |  |  | 10 f | 12 f |
|  | FLX | GRP-FLX | 10 m | 13 m |
|  |  |  | 11 f | 12 f |

**Supplementary Table S2.** Number and allocation of the offspring of socially isolated (SIR) and group-housed (GRP) dams treated either with vehicle (VEH) or fluoxetine (FLX). m, male; f, female. The number of animals included in each group was based on previous studies conducted in our laboratory assessing behavioral outcomes of prenatal manipulations.

***Behavioral testing***

In the dams, behavioral testing occurred after 5 and 10 weeks of the social isolation regimen, while offspring were tested once they reached adulthood (PND70). Behavioral testing was conducted by an experimenter that was blind to experimental groups. Socially isolated and group-housed dams were subjected, at 5 weeks, to the light-dark box test and the social interaction test to assess anxiety-like and social behavior, and to sampling of basal CORT (see below). At 10 weeks, they were subjected once again to the social interaction test and to the open field test to assess whether chronic treatment with FLX affects their performance in tests indexing social and anxiety-like behavior, respectively. The open field test was performed instead of the light dark box test at this time point to rule out any possible confounding factors due to pre-exposure to the light-dark box testing apparatus. Indeed, the latency to shuttle to the bright compartment in the light-dark box is extremely sensitive to the animals’ familiarity with the testing context, and repetition of the test could greatly affect the animal’s performance in the subsequent testing round. A separate cohort of animals, which were not used for subsequent breeding, were further subjected to basal measures of CORT one day before the FST, then to the FST, and then plasma CORT measurements 30’ after completion of the FST.

*Light-dark box test*

The light-dark box test was conducted using 4 identical Multi-Conditioning boxes (Multi Conditioning System, TSE, Germany). Each box contained a dark (1 Lux) and a bright (100 Lux) compartments, which were separated from each other by a dark plexiglass wall, within which there was an electrically controlled door. Each mouse was placed in the dark compartment to start the test. After a 5-sec acclimatization period, the door automatically opened, allowing the animals free access to both the dark and bright compartments for 5 minutes. The measurements collected from this test included the latency to move from the dark to the light compartment for the first time.

*Social interaction test*

Social interaction was assessed by analyzing the relative exploration time between an unfamiliar [congenic mouse](https://www.sciencedirect.com/topics/immunology-and-microbiology/congenic-strain) and an inanimate dummy object using methods established before^7^. The test apparatus was made of Plexiglas and consisted of three identical arms (50 cm × 9 cm; length × width) surrounded by 10-cm high Plexiglas walls. The three arms radiated from a central triangle (8 cm on each side) and spaced 120° from each other. Two out of the three arms contained a rectangular wire grid cage (13 cm × 8 cm × 10 cm, length × width × height; bars horizontally and vertically spaced 9 mm apart). The third arm did not contain a metal wire cage and served as the start zone (see below).

During the test phase, one metal wire cage contained an unfamiliar C57BL6/N mouse of the same sex and age, whereas the other wire cage contained an inanimate dummy object. The latter was a black scrunchie made of velvet material. The allocation of the unfamiliar live mouse and inanimate dummy object to the two wire cages was counterbalanced across experimental groups. To start a test trial, the test mouse was gently placed in the start arm and allowed to explore freely for 5 min. Behavioral observations were made by an experimenter blind to the experimental conditions, and social interaction was defined as nose contact within a 2-cm interaction zone. The relative time spent with the live mouse was calculated by the formula ([time spent with the mouse]/[time spent with the inanimate object + time spent with the mouse]) × 100 and used to compare the relative exploration time between the unfamiliar mouse and the inanimate dummy object. The total distance moved during the test was also measured to analyze general exploratory activity. This was achieved by a digital camera mounted above the apparatus, which provided images at a rate of 5 Hz that were transmitted to a PC running the EthoVision tracking system (Noldus [Information Technology](https://www.sciencedirect.com/topics/psychology/information-technology), The Netherlands).

*Open field test*

The test was conducted using 4 identical open field arenas (40 × 40 × 35-cm high) made of white plastic as described in^8-10^. They were located in a testing room under dim diffused lighting (approximately 35 lux as measured in the center of the arenas). A digital camera was mounted directly above the 4 arenas. Images were captured at a rate of 5 Hz and transmitted to a PC running the Ethovision (Noldus, Wageningen, The Netherlands) tracking system to record locomotor activity indexed by the distance moved in the entire open field arena. To start a trial, the animals were placed into the center of the open field arena and allowed to explore freely for 30 min. At the end of this time period, the animals were removed from the apparatus and returned to their home cage.

*Forced Swim test*

The forced swim test was adapted from previous protocols^7^. Mice were individually placed in a transparent Plexiglas cylinder (20 cm diameter), which was filled with water (21 ± 1°C) to a depth of 20 cm. The test lasted 6 minutes, and floating behavior was recorded during the last 4 min of the testing period.^7^ Each mouse was judged to be immobile when it ceased struggling and remained floating motionless in the water, making only those movements necessary to keep its head above water. At the end of the forced swim session, the animals were removed from the cylinder, gently dried with a paper towel, and placed in a fresh temporary cage for 30 minutes, after which time the mice were sacrificed. A digital camera was mounted directly above the water bath. Images were captured at a rate of 5 Hz and transmitted to a PC running the Ethovision (Noldus, Wageningen, The Netherlands) tracking system to record locomotor activity.

*Y-maze spatial recognition test*

The test apparatus was made of Plexiglas and consisted of three identical arms (50 cm × 9 cm; length × width) surrounded by 10-cm high Plexiglas walls. The three arms radiated from a central triangle (8 cm on each side) and spaced 120° from each other.

During the first phase of the test (sample phase), the mice were placed in the start arm, with one of the other two arms being blocked by an opaque plexiglass divider. Hence, during this phase, the mice were allowed to freely explore two arms of the Y-maze for 5 min before being removed and placed into a waiting box for 1 min.

For the second phase of the test (choice phase), the plexiglass divider was removed, and the animals were gently placed again into the start arm and were left to freely explore the entire Y-maze for 5 min. The relative time spent in the novel (previously blocked) arm was calculated by the formula ([time spent in the novel arm]/[time spent in familiar arm 1 + time spent in familiar arm 2+ time spent in unfamiliar arm]) × 100 and was used to compare the relative time spent exploring the novel arm and the familiar arms. The total distance moved during the test was also measured to analyze general exploratory activity. This was achieved by a digital camera mounted above the apparatus, which provided images at a rate of 5 Hz that were transmitted to a PC running the EthoVision tracking system (Noldus [Information Technology](https://www.sciencedirect.com/topics/psychology/information-technology), The Netherlands).

*Temporal order memory test*

The temporal order memory test was conducted in 4 identical open-field arenas (40 × 40 × 35-cm high) made of white plastic as described in^8-10^. They were located in a testing room under dim diffused lighting (approximately 35 lux as measured in the center of the arenas). A digital camera was mounted directly above the 4 arenas. Images were captured at a rate of 5 Hz and transmitted to a PC running the Ethovision (Noldus, Wageningen, The Netherlands) tracking system to record locomotor activity indexed by the distance moved in the entire open field arena.

During the first phase of the test, the animals were gently placed in the center of the open field arena and allowed to freely explore the arena for 10 min. They were then removed from the apparatus and placed in a holding room for 30 min before the start of the next phase. For the second phase of the test, a pair of two identical objects were placed in the open field arena in opposing corners (approximately 5 cm from the walls). The animals were placed back to the open field arena and allowed to freely interact with the objects for 10 min, after which they were again removed and placed in the holding room for another 30 min. Subsequently, a second pair of two identical objects, which differed from the pair of objects used previously, was placed into the open field. They were placed in the same position as the first pair of objects. The animals were placed again into the open field arena and allowed to explore the second pair of objects for 10 min, after which they were moved to the holding room once more for 30 min. For the final test phase, the open filed arena was equipped with one object from the first object pair (temporally more remote) and one object from the second object pair (temporally more recent). Again, the two objects were positioned in opposing corners, approximately 5 cm from the walls. For the final test phase, the animals were placed into the open field and allowed to freely explore the two objects for 10 min. Temporal order memory was indexed by analyzing the percentage time spent interacting with the temporally remote object during the test phase: ([time spent interacting with the remote object]/[time spent interacting with the remote object + time spent interacting with the recent object]) × 100.

***Corticosterone measurements***

Blood samples were taken from the tail vein of the mice as described previously^3^. Basal CORT sampling was performed between 2 and 5 hrs after onset of the dark-phase, which for nocturnal animals such as mice corresponds roughly to the peak of daily CORT^11^. The exact time of sampling was counterbalanced across groups. When considering the timing of matched basal and post-FST testing, the post-FST CORT samples were collected at the same circadian time point as the baseline data for each animal, and CORT collection and testing was again performed between 1.30 and 5 hrs after onset of the dark phase. For example, if animal A was tested for baseline CORT at 13 pm, the FST testing schedule was such so that the same animal was tested for post-FST CORT at 13pm on the day of the test. Moreover, the groups were counterbalanced so that no whole group was consistently tested at the beginning or at the end of the testing window, but rather they were all consistently spread out over the different testing times. Importantly, differences in plasma CORT measures were not influenced by the estrous cycle as measured by visual vaginal observation^12^ (*data not shown*). Plasma was collected after centrifugation at 10,000g at 4°C for 10 minutes and then stored at -20°C until further analysis. CORT was measured using a DirectX corticosterone enzyme immunoassay kit (Arbor Assays, Ann Arbor, MI, USA) following the manufacturer’s instructions.

***RNA extraction and next-generation RNA sequencing***

The adult offspring were killed by decapitation three weeks after completion of behavioral testing. The brains were rapidly extracted from the skull (within < 20 s) and placed on an ice-chilled plate. Coronal sections were prepared using razorblade cuts along the following coordinates with respect to bregma: anterior–posterior +2.0 to +1.0, +1.0 to 0.0, 0.0 to −1.0, −1.0 to −2.0, and −2.0 to −3.0 mm. Discrete brain regions were then collected using a micropunch needle (1 mm in diameter) generating micropunches of distinct brain areas as described previously^7^. Specifically, the micro-punching of the amygdala encompassed the collection of the central and basolateral nuclei with reference to bregma (-1.2 to -2.2 mm).

mRNA was extracted with the SPLIT kit (Lexogen, Germany) according to manufacturer’s recommendations. 5 animals from each group were included in the next-generation RNA sequencing (RNAseq) analysis. RNA integrity and amount were determined using a Bioanalyzer (Agilent). Only samples with an RNA integrity number > 8 were further processed and included in the analysis.

The Universal Plus mRNA kit (Nugen) preparation kit was used to produce library constructs. In brief, 500 ng of total RNA per sample was polyA enriched, fragmented and reverse transcribed into double-stranded cDNA and ligated with adapters. PCR was performed to selectively enrich for fragments containing adapters on both ends. Quality and quantity of enriched libraries were analyzed using Bioanalyzer 2100 (Agilent Technologies) with DNA-specific chip. The DNA library contained fragments in the size range of 100-500 base pairs (bp) with a mean fragment size of approximately 260 bp. Diluted libraries (10 nm) were pooled and sequenced on an Illumina NovaSeq 6000 to an average depth of ~20 million 100 bp single end reads per sample. Raw data is publicly accessible at the European Nucleotide Archive database under accession number PRJEB41610.

***RNA quality control and differential gene expression analyses***

Reads were quality-checked using FastQC. Low-quality ends were clipped (5’: 4 bases; 3’: 4 bases) and sequencing adaptors removed using Trimmomatic. Trimmed reads were mapped to the mouse genome (GRCm38.p6) and gene-level quantifications were calculated with Kallisto using the SUSHI data analysis framework^13^ with additional parameters, requiring at least 30bp matching, accepting at most 10 mismatches, and at most 5% of mismatches^14^. Quality control metrics were calculated on BAM files with SUSHI. Differential expression was computed for pairwise comparisons using the Bioconductor package EdgeR with false discovery rate (FDR)-corrected p-values (p < 0.05) set to a 5% threshold (q < 0.05), and an uncorrected p-value < 0.005.

Functional network prediction was generated through the use of QIAGEN’s Ingenuity Pathway Analysis (IPA, QIAGEN Redwood City). IPA uses the curated Ingenuity Knowledge Base to identify the involvement of differentially expressed genes in specific diseases and cellular pathways, and to establish functional networks of direct and indirect interactions between differentially expressed genes based on a functional analysis algorithm. For IPA, we used the differentially expressed genes identified by the Bioconductor package EdgeR with p-values (p < 0.005) as describe above. When considering networks and pathways, entities not associated with the central nervous system were excluded.

***Quantitative Real-Time PCR Analyses***

RNA was analyzed by TaqMan qRT-PCR instrument (CFX384 real-time system, Bio-Rad Laboratories) using the iScript one-step RT-PCR kit for probes (Bio-Rad Laboratories). The samples were run in 384-well formats in triplicates as multiplexed reactions with a normalizing internal control (36B4). We choose 36B4 as internal standard for gene expression analyses since its expression was not affected by the prenatal treatments^15^.

Thermal cycling was initiated with an incubation at 50°C for 10 min (RNA retrotranscription) and then at 95°C for 5 min (TaqMan polymerase activation). After this initial step, 39 cycles of PCR were performed. Each PCR cycle consisted of heating the samples at 95°C for 10 s to enable the melting process and then for 30 s at 60°C for the annealing and extension reaction. Relative target gene expression was calculated according to the 2(-Delta Delta C(T)) method^16^. Custom-designed probe and primer sequences, or product codes, used for the various genes of interest and reference gene (36B4) are summarized below and were purchased from Eurofins Genomics GmbH (Germany) and from Thermo Fisher Scientific (Germany).

| **Gene** | **Forward primer** | **Reverse primer** | **Probe** |
| --- | --- | --- | --- |
| Bdnf | 5’-AAGTCTGCATTACATTCCTCGA-3’ | 5’-GTTTTCTGAAAGAGGGACAGTTTAT-3’ | 5’-TGTGGTTTGTTGCCGTTGCCAAG-3’ |
| Reln | 5’-GGGTATAATCGGAATGTCTGGG-3’ | 5’-AGTAGAAAACTCCAAGCTGACG-3’ | 5’-TGTTGAAGGGAGAACGCGCAGC-3’ |
| Npas4 | 5’-GTCCTAATCTACCTGGGCTTTG-3’ | 5’-TCTCCACTTTCAGCCAACAG-3’ | 5’-ATGGTATGGACTGCTACACCCCGA-3’ |
| 36b4 | 5’-AGATGCAGCAGATCCGCAT-3’ | 5’-GTTCTTGCCCATCAGCACC-3’ | 5’-CGCTCCGAGGGAAGGCCG-3’ |
| **Gene** | **Product code** | | |
| Grin2a | Mm00433802_m1 | | |
| Grin2b | Mm00433820_m1 | | |
| Htr2A | Mm00555764_m1 | | |
| C3 | Mm00437863_m1 | | |
| Drd1 | Mm02620146_s1 | | |
| Drd2 | Mm00438545_m1 | | |
| Crhr2 | Mm00438308_m1 | | |
| S100b | Mm00485897_m1 | | |
| Rbp4 | Mm00803264_g1 | | |

**Supplementary Table S3.** Custom-designed probe and primer sequence (Eurofins), or product codes (Thermo Fisher Scientific), of the genes investigated with quantitative Real-Time PCR analyses.

***Statistical analyses***

All data met the assumptions of normal distribution and equality of variance; and all data were analyzed using parametric analysis of variance (ANOVA) or student’s t-test. Whenever appropriate, ANOVAs were followed by Tukey’s post-hoc test to control for multiple comparisons. All statistical analyses were performed using SPSS Statistics (version 22.0, IBM, Armonk, NY, USA) and Prism (version 7.0; GraphPad Software, La Jolla, CA, USA). Statistical significance was set at *p* < 0.05.

After 5 weeks of SIR or GRP, plasma CORT levels and data from the light-dark box and social interaction test were analyzed using student’s *t*-test. Weight gain over the first 7 weeks of SIR relative to GRP (i.e. before initiating maternal) FLX treatment was analyzed with a repeated-measure ANOVA (housing x sampling interval). After 10 weeks of SIR or GRP and additional FLX or VEH treatment, all data obtained from the dams were analyzed using two-way ANOVA (housing x treatment), except plasma CORT levels, which were analyzed using a 2 x 2 x 2 (sampling time x housing x treatment) repeated-measure ANOVA. All behavioral readouts in the offspring were analyzed using two-ways ANOVA (housing x treatment).

**Supplementary Figures and Tables**

**Supplementary Figure S1**

** A B C**

**Effects of maternal housing and treatment on litter size and sex distribution. (A)** The bar plots depict the litter size in the four different groups of dams. **(B)** The bar plots depict the number of male pups per litter in the four different groups of dams. **(C)** The bar plots depict the number of female pups per litter size in the four different groups of dams. No statistically significant effects were observed (N= 7-9 litters per group). FLX, fluoxetine; GRP, grouped-housed; SIR, social isolation rearing; VEH, vehicle.

**Supplementary Figure S2**

**Effects of maternal housing and treatment on pup weight at weaning.** **(A)** Body weight at weaning in female offspring. **(B)** Body weight at weaning in male offspring. No statistically significant effects were observed. N = 20-22 offspring per group and sex. FLX, fluoxetine; GRP, grouped-housed; SIR, social isolation rearing; VEH, vehicle

**Supplementary Figure S3**

**A B**

**Effects of maternal housing and treatment on weight gain in male and female offspring.** The line plots depict the weight gain in male and female offspring from weaning (PND 21) to adulthood (PND119). **(A)** In male offspring, maternal social isolation rearing (SIR) and fluoxetine (FLX) treatment both affected weight gain, leading to a significant main effect of maternal housing (*F*_(1,37)_ = 4.663, *p* < 0.05) and its interaction with FLX treatment (*F*_(1,37)_ = 7.060, *p <* 0.05). Subsequent post-hoc tests confirmed that male offspring born to SIR-VEH dams displayed increased weight gain compared to offspring born to GRP-VEH animals (PND98 SIR-VEH *vs* GRP-VEH °p < 0.05; PND105 SIR-VEH *vs* GRP-VEH °°p < 0.01; PND112 SIR-VEH *vs* GRP-VEH °p < 0.05; PND119 SIR-VEH *vs* GRP-VEH °p < 0.05) and to SIR-FLX offspring (PND105 SIR-FLX *vs* SIR-VEH ^§^p < 0.05; PND119 SIR-FLX *vs* SIR-VEH ^§^p < 0.05). **(B)** Maternal housing or drug treatment had no significant effect on weight gain in females.

**Supplementary Figure S4**

**Effects of maternal housing and treatment on total distance moved in the Y-maze spatial recognition test and in the temporal order test in male and female offspring.** **(A)** Total distance moved in the Y-maze test in male offspring **(B)** Total distance moved in the Y-maze test in female offspring. **(C)** Total distance moved in the temporal order memory test in male offspring. (D) Total distance moved in the temporal order memory test in female offspring. N= 10-11 mice per group and sex.

**Supplementary Figure S5**

**
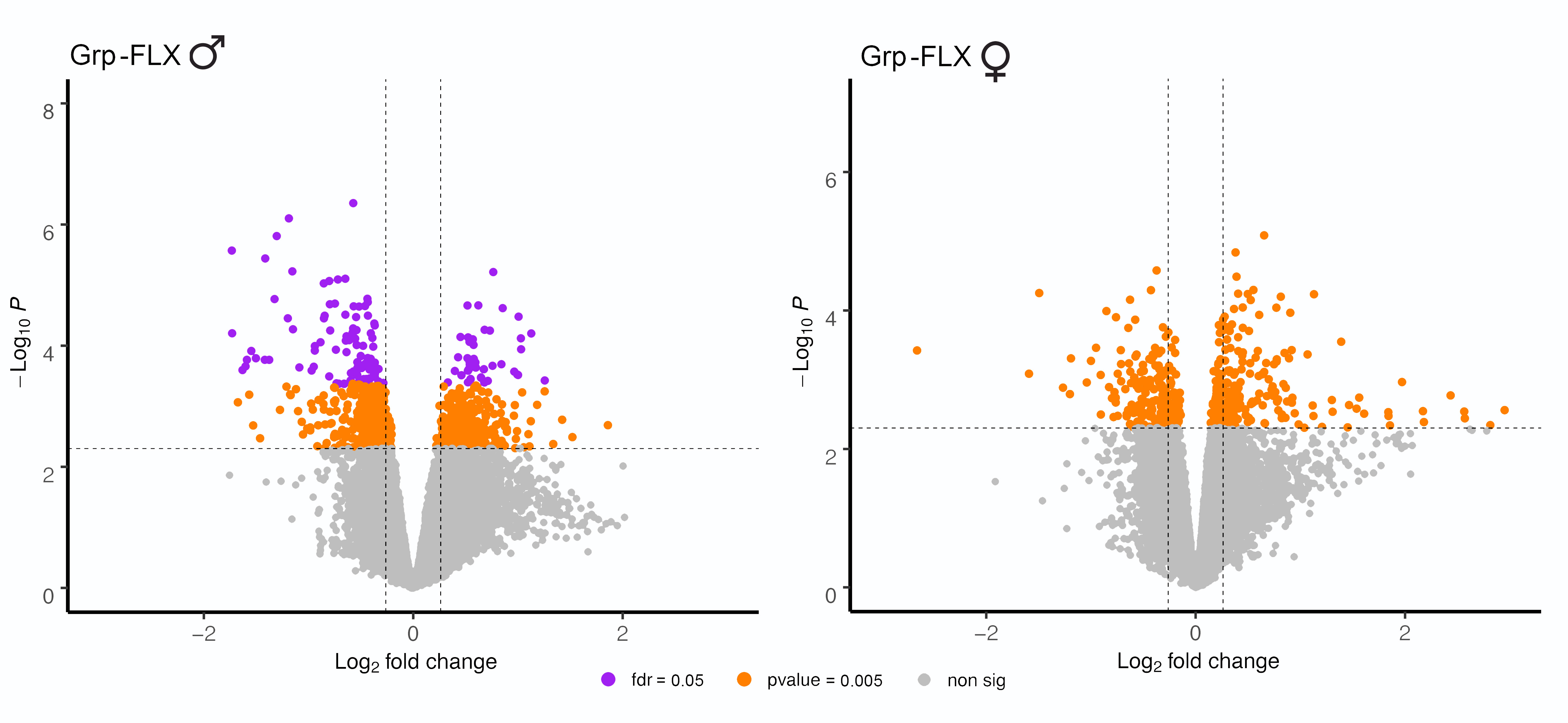
**

**Effects of maternal housing and treatment on the amygdalar transcriptome in male and female GRP-FLX offspring compared to GRP-VEH offspring**. The volcano plots depict differentially expressed genes (DEGs), including both changes that passed an FDR correction <0.05, and suggestive associations characterized by p < 0.005. In males, we uncovered 691 DEGs (135 q<0.05; 556 p<0.005), and in females 488 DEGs (0 q<0.05; 488 p<0.005). (N= 5 mice per group).

**Supplementary Figure S6**

**
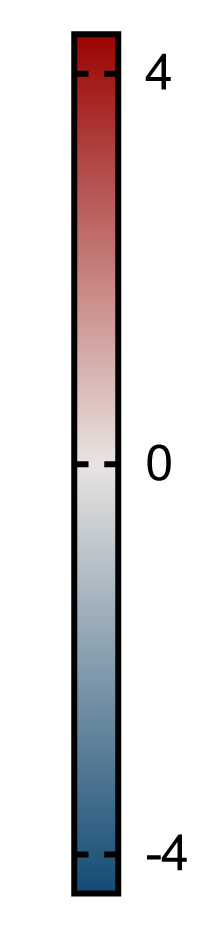
A B**

**Pathway analysis of differentially expressed genes in male and female GRP-FLX offspring relative to GRP-VEH offspring.** The bar plots depict the top 5 canonical pathways affected by the gene expression changes observed in male (A) and female (B) offspring. The color of the bar plots reflects the activation Z-score. White = Z-Score not available.

**Supplementary Figure S7**

**Validation of RNAseq gene expression changes by quantitative real-time PCR.** The bar plots depict mRNA levels in GRP-VEH, SIR-VEH and SIR-FLX male (blue hues) and female (rust-colored hues) offspring, respectively. The genes chosen are part of the biological function ‘memory’, and include Bdnf, Reln, Drd1, Drd2, Grin2a, Grin2b, C3, Npas4, Crhr2, s100b, Htr2a and Rbp4. The mRNA levels are reported as 2^^(-deltaCT)^. Appropriate post-hoc tests after significant main effects of one-way ANOVAs are the following: SIR-VEH *vs* GRP-VEH *p<0.05; **p<0.01; ***p<0.001; SIR-FLX *vs* SIR-VEH ^$^p<0.05; ^$$^p<0.01; ^$$$^p<0.001; SIR-FLX *vs* GRP-VEH °p<0.05; °°p<0.01; (N= 10-11 mice per group).

**Supplementary Figure S8**

**Heatmap depicting RT-qPCR mRNA expression changes.** The heatmap depicts mRNA expression changes investigated with quantitative real-time PCR in GRP-VEH, SIR-VEH and SIR-FLX male and female offspring. The selected genes are the same as depicted in **Supplementary Figure S4**, but for additional clarity, the data are expressed as SIR-VEH *vs* GRP-VEH and SIR-FLX *vs* SIR-VEH to match the data generated by the IPA analyses (see main Figure 4 and 5). The color-coded key denotes the level of downregulation (blue tones) or upregulation (magenta tones) of gene expression. N= 10-11 mice per group and sex.

**Supplementary Table S4**

|  | | | **SIR-VEH over GRP-VEH** | | |
| --- | --- | --- | --- | --- | --- |
| **Pathway** | **Gene symbol** | **Gene name** | ***p*-value** | ***q-*value** | **Log2FC** |
| **Calcium signaling** | ATP2B1 | ATPase plasma membrane Ca2+ transporting 1 | 3.53E-04 | 2.82E-02 | -0.428 |
|  | CACNA1A | calcium voltage-gated channel subunit alpha1 A | 1.03E-03 | 4.87E-02 | -0.257 |
|  | CACNA1C | calcium voltage-gated channel subunit alpha1 C | 1.28E-03 | 5.28E-02 | -0.237 |
|  | CACNA1D | calcium voltage-gated channel subunit alpha1 D | 1.22E-03 | 5.19E-02 | -0.252 |
|  | CACNB3 | calcium voltage-gated channel auxiliary subunit beta 3 | 4.47E-03 | 9.56E-02 | -0.272 |
|  | CAMK4 | calciumcalmodulin dependent protein kinase IV | 5.19E-04 | 3.53E-02 | -0.408 |
|  | CAMK1G | calciumcalmodulin dependent protein kinase IG | 1.09E-03 | 4.94E-02 | 0.327 |
|  | CAMK2B | calciumcalmodulin dependent protein kinase II beta | 1.76E-03 | 5.95E-02 | -0.34 |
|  | CAMKK1 | calciumcalmodulin dependent protein kinase kinase 1 | 4.93E-03 | 1.00E-01 | -0.264 |
|  | CAMKK2 | calciumcalmodulin dependent protein kinase kinase 2 | 5.41E-05 | 1.12E-02 | -0.486 |
|  | CHRNA5 | cholinergic receptor nicotinic alpha 5 subunit | 4.36E-03 | 9.43E-02 | -0.652 |
|  | GRIN2A | glutamate ionotropic receptor NMDA type subunit 2A | 8.80E-05 | 1.39E-02 | -0.376 |
|  | GRIN2B | glutamate ionotropic receptor NMDA type subunit 2B | 2.09E-03 | 6.35E-02 | -0.242 |
|  | GRIN2D | glutamate ionotropic receptor NMDA type subunit 2D | 1.62E-03 | 5.80E-02 | 0.357 |
|  | HDAC11 | histone deacetylase 11 | 1.17E-04 | 1.59E-02 | 0.26 |
|  | ITPR1 | inositol 1,4,5-trisphosphate receptor type 1 | 1.35E-03 | 5.38E-02 | -0.529 |
|  | MYH3 | myosin heavy chain 3 | 3.47E-05 | 8.79E-03 | -1.118 |
|  | PNCK | pregnancy up-regulated nonubiquitous CaM kinase | 1.26E-04 | 1.61E-02 | 0.385 |
|  | PPP3CA | protein phosphatase 3 catalytic subunit alpha | 3.94E-03 | 9.07E-02 | -0.368 |
|  | PPP3R1 | protein phosphatase 3 regulatory subunit B, alpha | 1.94E-04 | 2.09E-02 | -0.324 |
|  | PRKAG2 | protein kinase AMP-activated non-catalytic subunit gamma 2 | 6.68E-04 | 3.87E-02 | -0.274 |
|  | RCAN3 | RCAN family member 3 | 1.55E-03 | 5.66E-02 | 0.326 |
|  | SLC8A2 | solute carrier family 8 member A2 | 9.27E-05 | 1.41E-02 | -0.384 |
|  | Tpm1 | tropomyosin 1, alpha | 9.57E-04 | 4.68E-02 | -0.345 |
|  | TRPC1 | transient receptor potential cation channel subfamily C member 1 | 4.05E-03 | 9.18E-02 | -0.273 |
|  | TRPC5 | transient receptor potential cation channel subfamily C member 5 | 3.06E-03 | 7.73E-02 | 0.428 |
|  | TRPC6 | transient receptor potential cation channel subfamily C member 6 | 3.11E-03 | 7.80E-02 | -0.51 |
| **Endocannabinoid signaling** | ADCY1 | adenylate cyclase 1 | 3.21E-04 | 2.73E-02 | -0.442 |
|  | ADCY9 | adenylate cyclase 9 | 4.04E-05 | 9.61E-03 | -0.353 |
|  | CACNA1A | calcium voltage-gated channel subunit alpha1 A | 1.03E-03 | 4.87E-02 | -0.257 |
|  | CACNA1C | calcium voltage-gated channel subunit alpha1 C | 1.28E-03 | 5.28E-02 | -0.237 |
|  | CACNA1D | calcium voltage-gated channel subunit alpha1 D | 1.22E-03 | 5.19E-02 | -0.252 |
|  | CACNB3 | calcium voltage-gated channel auxiliary subunit beta 3 | 4.47E-03 | 9.56E-02 | -0.272 |
|  | CNR1 | cannabinoid receptor 1 | 4.95E-03 | 1.01E-01 | -0.222 |
|  | DAGLA | diacylglycerol lipase alpha | 2.78E-03 | 7.34E-02 | -0.308 |
|  | GNAI2 | G protein subunit alpha i2 | 7.50E-04 | 4.18E-02 | 0.248 |
|  | GRIN2A | glutamate ionotropic receptor NMDA type subunit 2A | 8.80E-05 | 1.39E-02 | -0.376 |
|  | GRIN2B | glutamate ionotropic receptor NMDA type subunit 2B | 2.09E-03 | 6.35E-02 | -0.242 |
|  | GRIN2D | glutamate ionotropic receptor NMDA type subunit 2D | 1.62E-03 | 5.80E-02 | 0.357 |
|  | ITPR1 | inositol 1,4,5-trisphosphate receptor type 1 | 1.35E-03 | 5.38E-02 | -0.529 |
|  | MAPK11 | mitogen-activated protein kinase 11 | 1.71E-04 | 1.96E-02 | -0.548 |
|  | MGLL | monoglyceride lipase | 4.18E-04 | 3.13E-02 | -0.308 |
|  | PPP3CA | protein phosphatase 3 catalytic subunit alpha | 3.94E-03 | 9.07E-02 | -0.368 |
|  | PPP3R1 | protein phosphatase 3 regulatory subunit B, alpha | 1.94E-04 | 2.09E-02 | -0.324 |
|  | PRKAG2 | protein kinase AMP-activated non-catalytic subunit gamma 2 | 6.68E-04 | 3.87E-02 | -0.274 |
|  | PTGS2 | prostaglandin-endoperoxide synthase 2 | 8.83E-04 | 4.53E-02 | -0.889 |
|  | RIMS1 | regulating synaptic membrane exocytosis 1 | 2.21E-04 | 2.24E-02 | -0.351 |
| **Synaptogenesis signaling pathway** | ADCY1 | adenylate cyclase 1 | 3.21E-04 | 2.73E-02 | -0.442 |
|  | ADCY9 | adenylate cyclase 9 | 4.04E-05 | 9.61E-03 | -0.353 |
|  | APOE | apolipoprotein E | 3.67E-03 | 8.76E-02 | 0.298 |
|  | BDNF | brain derived neurotrophic factor | 2.73E-05 | 7.82E-03 | -0.944 |
|  | CACNB3 | calcium voltage-gated channel auxiliary subunit beta 3 | 4.47E-03 | 9.56E-02 | -0.272 |
|  | CAMK4 | calciumcalmodulin dependent protein kinase IV | 5.19E-04 | 3.53E-02 | -0.408 |
|  | CAMK2B | calciumcalmodulin dependent protein kinase II beta | 1.76E-03 | 5.95E-02 | -0.34 |
|  | CDH11 | cadherin 11 | 4.18E-03 | 9.32E-02 | -0.278 |
|  | CDH23 | cadherin related 23 | 2.26E-03 | 6.60E-02 | 0.705 |
|  | CHN1 | chimerin 1 | 6.34E-04 | 3.79E-02 | -0.358 |
|  | CPLX2 | complexin 2 | 8.14E-04 | 4.33E-02 | -0.36 |
|  | CPLX3 | complexin 3 | 4.44E-05 | 9.94E-03 | -1.058 |
|  | DAB1 | DAB adaptor protein 1 | 4.05E-04 | 3.08E-02 | -0.371 |
|  | EFNB2 | ephrin B2 | 1.40E-03 | 5.41E-02 | -0.288 |
|  | EFNB3 | ephrin B3 | 3.74E-05 | 9.18E-03 | 0.455 |
|  | EPHB6 | EPH receptor B6 | 2.93E-04 | 2.60E-02 | -0.488 |
|  | GRIN2A | glutamate ionotropic receptor NMDA type subunit 2A | 8.80E-05 | 1.39E-02 | -0.376 |
|  | GRIN2B | glutamate ionotropic receptor NMDA type subunit 2B | 2.09E-03 | 6.35E-02 | -0.242 |
|  | GRIN2D | glutamate ionotropic receptor NMDA type subunit 2D | 1.62E-03 | 5.80E-02 | 0.357 |
|  | ITPR1 | inositol 1,4,5-trisphosphate receptor type 1 | 1.35E-03 | 5.38E-02 | -0.529 |
|  | KALRN | kalirin RhoGEF kinase | 4.46E-04 | 3.19E-02 | -0.39 |
|  | LRRTM2 | leucine rich repeat transmembrane neuronal 2 | 1.45E-03 | 5.52E-02 | -0.253 |
|  | PRKAG2 | protein kinase AMP-activated non-catalytic subunit gamma 2 | 6.68E-04 | 3.87E-02 | -0.274 |
|  | PRKCE | protein kinase C epsilon | 7.98E-04 | 4.29E-02 | -0.243 |
|  | RASGRP1 | RAS guanyl releasing protein 1 | 1.22E-03 | 5.19E-02 | -0.341 |
|  | SNAP25 | synaptosome associated protein 25 | 2.34E-03 | 6.74E-02 | -0.288 |
|  | STX1A | syntaxin 1A | 1.02E-03 | 4.85E-02 | -0.498 |
|  | SYNGAP1 | synaptic Ras GTPase activating protein 1 | 2.09E-03 | 6.35E-02 | -0.242 |
|  | SYT7 | synaptotagmin 7 | 1.98E-03 | 6.25E-02 | -0.241 |
|  | UNC13A | unc-13 homolog A | 5.95E-04 | 3.72E-02 | -0.287 |
|  | WASF1 | WASP family member 1 | 1.60E-03 | 5.74E-02 | -0.264 |
| **Opioid signaling pathway** | ADCY1 | adenylate cyclase 1 | 3.21E-04 | 2.73E-02 | -0.442 |
|  | ADCY9 | adenylate cyclase 9 | 4.04E-05 | 9.61E-03 | -0.353 |
|  | CACNA1A | calcium voltage-gated channel subunit alpha1 A | 1.03E-03 | 4.87E-02 | -0.257 |
|  | CACNA1C | calcium voltage-gated channel subunit alpha1 C | 1.28E-03 | 5.28E-02 | -0.237 |
|  | CACNA1D | calcium voltage-gated channel subunit alpha1 D | 1.22E-03 | 5.19E-02 | -0.252 |
|  | CACNB3 | calcium voltage-gated channel auxiliary subunit beta 3 | 4.47E-03 | 9.56E-02 | -0.272 |
|  | CAMK4 | calciumcalmodulin dependent protein kinase IV | 5.19E-04 | 3.53E-02 | -0.408 |
|  | CAMK1G | calciumcalmodulin dependent protein kinase IG | 1.09E-03 | 4.94E-02 | 0.327 |
|  | CAMK2B | calciumcalmodulin dependent protein kinase II beta | 1.76E-03 | 5.95E-02 | -0.34 |
|  | FOSB | FosB proto-oncogene, AP-1 transcription factor subunit | 2.49E-03 | 6.99E-02 | -0.848 |
|  | GNAI2 | G protein subunit alpha i2 | 7.50E-04 | 4.18E-02 | 0.248 |
|  | GRIN2A | glutamate ionotropic receptor NMDA type subunit 2A | 8.80E-05 | 1.39E-02 | -0.376 |
|  | GRIN2B | glutamate ionotropic receptor NMDA type subunit 2B | 2.09E-03 | 6.35E-02 | -0.242 |
|  | GRIN2D | glutamate ionotropic receptor NMDA type subunit 2D | 1.62E-03 | 5.80E-02 | 0.357 |
|  | ITPR1 | inositol 1,4,5-trisphosphate receptor type 1 | 1.35E-03 | 5.38E-02 | -0.529 |
|  | OPRL1 | opioid related nociceptin receptor 1 | 3.82E-03 | 8.91E-02 | 0.314 |
|  | PPP3CA | protein phosphatase 3 catalytic subunit alpha | 3.94E-03 | 9.07E-02 | -0.368 |
|  | PPP3R1 | protein phosphatase 3 regulatory subunit B, alpha | 1.94E-04 | 2.09E-02 | -0.324 |
|  | PRKAG2 | protein kinase AMP-activated non-catalytic subunit gamma 2 | 6.68E-04 | 3.87E-02 | -0.274 |
|  | PRKCB | protein kinase C beta | 1.63E-03 | 5.82E-02 | -0.313 |
|  | PRKCE | protein kinase C epsilon | 7.98E-04 | 4.29E-02 | -0.243 |
|  | RGS3 | regulator of G protein signaling 3 | 1.10E-03 | 4.98E-02 | 0.413 |
|  | RGS11 | regulator of G protein signaling 11 | 2.23E-03 | 6.55E-02 | -0.405 |
|  | RGS12 | regulator of G protein signaling 12 | 4.11E-04 | 3.10E-02 | -0.368 |
|  | RPS6KA1 | ribosomal protein S6 kinase A1 | 2.82E-03 | 7.37E-02 | 0.373 |
|  | RPS6KA3 | ribosomal protein S6 kinase A3 | 3.58E-05 | 8.91E-03 | -0.473 |
| **Dopamine-DARPP32 Feedback** | ADCY1 | adenylate cyclase 1 | 3.21E-04 | 2.73E-02 | -0.442 |
|  | ADCY9 | adenylate cyclase 9 | 4.04E-05 | 9.61E-03 | -0.353 |
|  | CACNA1A | calcium voltage-gated channel subunit alpha1 A | 1.03E-03 | 4.87E-02 | -0.257 |
|  | CACNA1C | calcium voltage-gated channel subunit alpha1 C | 1.28E-03 | 5.28E-02 | -0.237 |
|  | CACNA1D | calcium voltage-gated channel subunit alpha1 D | 1.22E-03 | 5.19E-02 | -0.252 |
|  | CAMK4 | calciumcalmodulin dependent protein kinase IV | 5.19E-04 | 3.53E-02 | -0.408 |
|  | CAMKK1 | calciumcalmodulin dependent protein kinase kinase 1 | 4.93E-03 | 1.00E-01 | -0.264 |
|  | CAMKK2 | calciumcalmodulin dependent protein kinase kinase 2 | 5.41E-05 | 1.12E-02 | -0.486 |
|  | GNAI2 | G protein subunit alpha i2 | 7.50E-04 | 4.18E-02 | 0.248 |
|  | GRIN2A | glutamate ionotropic receptor NMDA type subunit 2A | 8.80E-05 | 1.39E-02 | -0.376 |
|  | GRIN2B | glutamate ionotropic receptor NMDA type subunit 2B | 2.09E-03 | 6.35E-02 | -0.242 |
|  | GRIN2D | glutamate ionotropic receptor NMDA type subunit 2D | 1.62E-03 | 5.80E-02 | 0.357 |
|  | ITPR1 | inositol 1,4,5-trisphosphate receptor type 1 | 1.35E-03 | 5.38E-02 | -0.529 |
|  | PPP2R2C | protein phosphatase 2 regulatory subunit Bgamma | 2.49E-03 | 6.99E-02 | -0.223 |
|  | PPP2R3A | protein phosphatase 2 regulatory subunit B''alpha | 2.21E-04 | 2.24E-02 | 0.293 |
|  | PPP3CA | protein phosphatase 3 catalytic subunit alpha | 3.94E-03 | 9.07E-02 | -0.368 |
|  | PPP3R1 | protein phosphatase 3 regulatory subunit B, alpha | 1.94E-04 | 2.09E-02 | -0.324 |
|  | PRKAG2 | protein kinase AMP-activated non-catalytic subunit gamma 2 | 6.68E-04 | 3.87E-02 | -0.274 |
|  | PRKCB | protein kinase C beta | 1.63E-03 | 5.82E-02 | -0.313 |
|  | PRKCE | protein kinase C epsilon | 7.98E-04 | 4.29E-02 | -0.243 |

**List of genes annotated with the top five canonical signaling pathways affected in male SIR-VEH versus GRP-VEH offspring.** Ingenuity Pathway Analysis (IPA) was used to identify canonical signaling pathways affected in the amygdala of SIR-VEH male offspring relative to GRP-VEH offspring. The table lists the top five signaling pathways and the differentially expressed genes (DEGs) annotated with each pathway. The direction of transcriptional changes is shown in terms of log2 fold changes (Log2FC). The significance is reported both in terms of false discovery rate (FDR) correction set at a 5% threshold (*q* < 0.05) and of uncorrected p value.

**Supplementary Table S5**

|  | | | **SIR-FLX over SIR-VEH** | | |
| --- | --- | --- | --- | --- | --- |
| **Pathway** | **Gene symbol** | **Gene name** | ***p*-value** | ***q-*value** | **Log2FC** |
| **Axonal Guidance** | Adam1a | a disintegrin and metallopeptidase domain 1a | 1.82E-03 | 2.06E-01 | -0.35 |
|  | ARPC3 | actin related protein 23 complex subunit 3 | 2.41E-03 | 2.07E-01 | 0.222 |
|  | ARPC1B | actin related protein 23 complex subunit 1B | 3.19E-03 | 2.07E-01 | -0.397 |
|  | BDNF | brain derived neurotrophic factor | 6.99E-06 | 2.57E-02 | 0.615 |
|  | BMP3 | bone morphogenetic protein 3 | 2.78E-03 | 2.07E-01 | 0.249 |
|  | ECE2 | endothelin converting enzyme 2 | 1.44E-03 | 2.06E-01 | -0.287 |
|  | ECEL1 | endothelin converting enzyme like 1 | 2.20E-04 | 1.35E-01 | -0.526 |
|  | EFNB2 | ephrin B2 | 2.37E-04 | 1.40E-01 | 0.373 |
|  | EPHA7 | EPH receptor A7 | 1.36E-03 | 2.06E-01 | 0.321 |
|  | GNA12 | G protein subunit alpha 12 | 3.51E-03 | 2.08E-01 | -0.285 |
|  | GNB4 | G protein subunit beta 4 | 8.49E-05 | 9.41E-02 | 0.447 |
|  | KALRN | kalirin RhoGEF kinase | 3.67E-04 | 1.71E-01 | 0.298 |
|  | MMP17 | matrix metallopeptidase 17 | 1.62E-03 | 2.06E-01 | 0.349 |
|  | NTNG2 | netrin G2 | 5.52E-04 | 1.71E-01 | 0.442 |
|  | PLCH1 | phospholipase C eta 1 | 4.00E-03 | 2.14E-01 | -0.539 |
|  | PLXND1 | plexin D1 | 1.06E-03 | 2.03E-01 | 0.558 |
|  | PRKCD | protein kinase C delta | 1.82E-03 | 2.06E-01 | -1.043 |
|  | ROBO3 | roundabout guidance receptor 3 | 4.98E-03 | 2.24E-01 | 0.784 |
|  | SEMA6A | semaphorin 6A | 1.21E-03 | 2.06E-01 | -0.342 |
|  | UNC5B | unc-5 netrin receptor B | 3.57E-03 | 2.10E-01 | -0.463 |
|  | WIPF1 | WASWASL interacting protein family member 1 | 3.39E-03 | 2.07E-01 | -0.374 |
|  | WNT3 | Wnt family member 3 | 3.09E-03 | 2.07E-01 | -0.632 |
| **Actin Nucleation** | ARPC3 | actin related protein 23 complex subunit 3 | 2.41E-03 | 2.07E-01 | 0.222 |
|  | ARPC1B | actin related protein 23 complex subunit 1B | 3.19E-03 | 2.07E-01 | -0.397 |
|  | FNBP1 | formin binding protein 1 | 3.19E-03 | 2.07E-01 | -0.352 |
|  | GNA12 | G protein subunit alpha 12 | 3.51E-03 | 2.08E-01 | -0.285 |
|  | RHOG | ras homolog family member G | 3.34E-03 | 2.07E-01 | -0.407 |
|  | RHOQ | ras homolog family member Q | 2.39E-03 | 2.07E-01 | -0.262 |
|  | WASF1 | WASP family member 1 | 3.26E-03 | 2.07E-01 | 0.272 |
|  | WIPF1 | WASWASL interacting protein family member 1 | 3.39E-03 | 2.07E-01 | -0.374 |
| **Acute phase response signaling** | AGT | angiotensinogen | 2.91E-03 | 2.07E-01 | -0.644 |
|  | C4A/C4B | complement C4B (Chido blood group) | 5.98E-04 | 1.71E-01 | -0.666 |
|  | IL33 | interleukin 33 | 2.91E-03 | 2.07E-01 | -0.457 |
|  | IL1RAP | interleukin 1 receptor accessory protein | 4.47E-03 | 2.20E-01 | -0.405 |
|  | MAPK11 | mitogen-activated protein kinase 11 | 2.01E-03 | 2.06E-01 | 0.439 |
|  | MAPK13 | mitogen-activated protein kinase 13 | 3.75E-03 | 2.11E-01 | 0.564 |
|  | RBP4 | retinol binding protein 4 | 1.92E-04 | 1.28E-01 | 0.744 |
|  | SERPIND1 | serpin family D member 1 | 3.18E-03 | 2.07E-01 | -0.547 |
|  | SERPINF1 | serpin family F member 1 | 3.42E-03 | 2.07E-01 | -0.597 |
|  | SOCS7 | suppressor of cytokine signaling 7 | 2.60E-03 | 2.07E-01 | 0.208 |
|  | TNFRSF1A | TNF receptor superfamily member 1A | 2.77E-03 | 2.07E-01 | -0.376 |
| **Production of NO and ROS** | APOD | apolipoprotein D | 2.27E-03 | 2.07E-01 | -0.566 |
|  | FNBP1 | formin binding protein 1 | 3.19E-03 | 2.07E-01 | -0.352 |
|  | MAPK11 | mitogen-activated protein kinase 11 | 2.01E-03 | 2.06E-01 | 0.439 |
|  | MAPK13 | mitogen-activated protein kinase 13 | 3.75E-03 | 2.11E-01 | 0.564 |
|  | PPP2R3A | protein phosphatase 2 regulatory subunit B''alpha | 3.18E-03 | 2.07E-01 | -0.331 |
|  | PRKCD | protein kinase C delta | 1.82E-03 | 2.06E-01 | -1.043 |
|  | RBP4 | retinol binding protein 4 | 1.92E-04 | 1.28E-01 | 0.744 |
|  | RHOG | ras homolog family member G | 3.34E-03 | 2.07E-01 | -0.407 |
|  | RHOQ | ras homolog family member Q | 2.39E-03 | 2.07E-01 | -0.262 |
|  | TNFRSF1A | TNF receptor superfamily member 1A | 2.77E-03 | 2.07E-01 | -0.376 |
| **Synaptogenesis signaling pathway** | ARPC3 | actin related protein 23 complex subunit 3 | 2.41E-03 | 2.07E-01 | 0.222 |
|  | ARPC1B | actin related protein 23 complex subunit 1B | 3.19E-03 | 2.07E-01 | -0.397 |
|  | BDNF | brain derived neurotrophic factor | 6.99E-06 | 2.57E-02 | 0.615 |
|  | CACNB3 | calcium voltage-gated channel auxiliary subunit beta 3 | 3.58E-03 | 2.10E-01 | 0.351 |
|  | CHN1 | chimerin 1 | 1.02E-03 | 2.01E-01 | 0.374 |
|  | EFNB2 | ephrin B2 | 2.37E-04 | 1.40E-01 | 0.373 |
|  | EPHA7 | EPH receptor A7 | 1.36E-03 | 2.06E-01 | 0.321 |
|  | KALRN | kalirin RhoGEF kinase | 3.67E-04 | 1.71E-01 | 0.298 |
|  | PRKCD | protein kinase C delta | 1.82E-03 | 2.06E-01 | -1.043 |
|  | STX1A | syntaxin 1A | 6.17E-04 | 1.71E-01 | 0.548 |
|  | SYT17 | synaptotagmin 17 | 3.69E-03 | 2.11E-01 | 0.409 |
|  | UNC13A | unc-13 homolog A | 2.06E-03 | 2.06E-01 | 0.27 |
|  | WASF1 | WASP family member 1 | 3.26E-03 | 2.07E-01 | 0.272 |

**List of genes annotated with the top five canonical signaling pathways affected in male SIR-FLX versus SIR-VEH offspring.** Ingenuity Pathway Analysis (IPA) was used to identify canonical signaling pathways affected in the amygdala of SIR-FLX male offspring relative to SIR-VEH male offspring. The table lists the top five signaling pathways and the differentially expressed genes (DEGs) annotated with each pathway. The direction of transcriptional changes is shown in terms of log2 fold changes (Log2FC). The significance is reported both in terms of false discovery rate (FDR) correction set at a 5% threshold (*q* < 0.05) and of uncorrected p value.

**Supplementary Table S6**

|  | | | **SIR-VEH over GRP-VEH** | | |
| --- | --- | --- | --- | --- | --- |
| **Pathway** | **Gene symbol** | **Gene name** | ***p*-value** | ***q-*value** | **Log2FC** |
| **Opioid Signaling pathway** | ADCY5 | adenylate cyclase 5 | 5.24E-04 | 1.79E-02 | -0.691 |
|  | ADCY6 | adenylate cyclase 6 | 2.31E-03 | 3.88E-02 | 0.323 |
|  | AP2S1 | adaptor related protein complex 2 subunit sigma 1 | 3.48E-03 | 4.79E-02 | 0.189 |
|  | BAD | BCL2 associated agonist of cell death | 3.13E-03 | 4.51E-02 | 0.234 |
|  | CACNA1H | calcium voltage-gated channel subunit alpha1 H | 2.08E-03 | 3.68E-02 | -0.292 |
|  | CACNA1I | calcium voltage-gated channel subunit alpha1 I | 3.30E-04 | 1.43E-02 | -0.424 |
|  | CACNA2D1 | calcium voltage-gated channel auxiliary subunit alpha2delta 1 | 3.05E-03 | 4.45E-02 | 0.348 |
|  | CACNA2D2 | calcium voltage-gated channel auxiliary subunit alpha2delta 2 | 3.08E-04 | 1.38E-02 | -0.399 |
|  | CACNA2D3 | calcium voltage-gated channel auxiliary subunit alpha2delta 3 | 5.05E-04 | 1.75E-02 | -0.586 |
|  | CACNB2 | calcium voltage-gated channel auxiliary subunit beta 2 | 1.32E-03 | 2.88E-02 | -0.339 |
|  | CACNG4 | calcium voltage-gated channel auxiliary subunit gamma 4 | 4.74E-04 | 1.70E-02 | -0.319 |
|  | CAMK2B | calciumcalmodulin dependent protein kinase II beta | 4.11E-03 | 5.23E-02 | -0.268 |
|  | CTNNB1 | catenin beta 1 | 3.77E-03 | 5.00E-02 | 0.162 |
|  | GNAI1 | G protein subunit alpha i1 | 1.48E-03 | 3.07E-02 | 0.18 |
|  | GNAL | G protein subunit alpha L | 3.26E-04 | 1.42E-02 | -0.558 |
|  | GNG7 | G protein subunit gamma 7 | 5.10E-04 | 1.75E-02 | -0.822 |
|  | GNG11 | G protein subunit gamma 11 | 6.60E-04 | 2.04E-02 | -0.441 |
|  | GRK3 | G protein-coupled receptor kinase 3 | 2.89E-03 | 4.31E-02 | 0.294 |
|  | KCNJ3 | potassium inwardly rectifying channel subfamily J member 3 | 8.84E-04 | 2.39E-02 | 0.264 |
|  | KCNJ6 | potassium inwardly rectifying channel subfamily J member 6 | 2.14E-03 | 3.71E-02 | 0.506 |
|  | MAPK3 | mitogen-activated protein kinase 3 | 3.11E-03 | 4.50E-02 | 0.238 |
|  | MAPK6 | mitogen-activated protein kinase 6 | 1.25E-03 | 2.85E-02 | -0.263 |
|  | MAPK15 | mitogen-activated protein kinase 15 | 9.46E-04 | 2.44E-02 | 0.922 |
|  | PDE1A | phosphodiesterase 1A | 4.67E-03 | 5.56E-02 | 0.447 |
|  | PDE1B | phosphodiesterase 1B | 2.91E-04 | 1.35E-02 | -0.821 |
|  | PDE1C | phosphodiesterase 1C | 2.41E-03 | 3.93E-02 | -0.503 |
|  | PDK1 | pyruvate dehydrogenase kinase 1 | 6.01E-04 | 1.94E-02 | 0.458 |
|  | PDYN | prodynorphin | 1.98E-04 | 1.14E-02 | -0.568 |
|  | PENK | proenkephalin | 1.74E-04 | 1.07E-02 | -0.724 |
|  | PLD2 | phospholipase D2 | 1.24E-03 | 2.84E-02 | -0.248 |
|  | PRKCD | protein kinase C delta | 2.80E-04 | 1.33E-02 | -1.24 |
|  | PRKCH | protein kinase C eta | 2.56E-04 | 1.29E-02 | -0.723 |
|  | PRKD1 | protein kinase D1 | 4.18E-04 | 1.58E-02 | -0.538 |
|  | PRKD3 | protein kinase D3 | 4.73E-04 | 1.70E-02 | -0.329 |
|  | RAC3 | Rac family small GTPase 3 | 2.47E-03 | 3.98E-02 | 0.331 |
|  | RASD2 | RASD family member 2 | 1.93E-04 | 1.13E-02 | -1.073 |
|  | RGS4 | regulator of G protein signaling 4 | 1.31E-04 | 9.43E-03 | -0.678 |
|  | RGS8 | regulator of G protein signaling 8 | 1.42E-03 | 2.99E-02 | -0.419 |
|  | RGS9 | regulator of G protein signaling 9 | 1.35E-04 | 9.52E-03 | -1.256 |
|  | RPS6KA5 | ribosomal protein S6 kinase A5 | 1.34E-03 | 2.89E-02 | -0.319 |
|  | RYR3 | ryanodine receptor 3 | 9.18E-05 | 8.27E-03 | -0.479 |
| **Axonal guidance signaling** | ACE | angiotensin I converting enzyme | 8.21E-06 | 3.27E-03 | 1.955 |
|  | ADAM23 | ADAM metallopeptidase domain 23 | 4.60E-04 | 1.68E-02 | -0.364 |
|  | ADAM33 | ADAM metallopeptidase domain 33 | 1.14E-03 | 2.71E-02 | 0.718 |
|  | ADAMTS3 | ADAM metallopeptidase with thrombospondin type 1 motif 3 | 2.01E-03 | 3.62E-02 | -0.526 |
|  | ADAMTS4 | ADAM metallopeptidase with thrombospondin type 1 motif 4 | 4.23E-04 | 1.58E-02 | -0.473 |
|  | ARHGEF15 | Rho guanine nucleotide exchange factor 15 | 5.97E-04 | 1.93E-02 | -0.466 |
|  | BDNF | brain derived neurotrophic factor | 3.36E-03 | 4.67E-02 | 0.714 |
|  | BMP6 | bone morphogenetic protein 6 | 1.96E-03 | 3.55E-02 | 1.016 |
|  | BMP7 | bone morphogenetic protein 7 | 2.34E-03 | 3.89E-02 | 0.684 |
|  | EFNB1 | ephrin B1 | 4.43E-03 | 5.39E-02 | 0.451 |
|  | EPHA3 | EPH receptor A3 | 3.24E-03 | 4.60E-02 | 0.497 |
|  | EPHA8 | EPH receptor A8 | 2.73E-03 | 4.20E-02 | -0.576 |
|  | EPHB1 | EPH receptor B1 | 5.43E-05 | 6.47E-03 | -0.547 |
|  | FZD4 | frizzled class receptor 4 | 2.64E-04 | 1.32E-02 | 0.771 |
|  | FZD8 | frizzled class receptor 8 | 5.03E-04 | 1.75E-02 | 0.461 |
|  | GLI3 | GLI family zinc finger 3 | 1.97E-03 | 3.57E-02 | -0.411 |
|  | GNA12 | G protein subunit alpha 12 | 3.72E-04 | 1.49E-02 | -0.268 |
|  | GNAI1 | G protein subunit alpha i1 | 1.48E-03 | 3.07E-02 | 0.18 |
|  | GNAL | G protein subunit alpha L | 3.26E-04 | 1.42E-02 | -0.558 |
|  | GNG7 | G protein subunit gamma 7 | 5.10E-04 | 1.75E-02 | -0.822 |
|  | GNG11 | G protein subunit gamma 11 | 6.60E-04 | 2.04E-02 | -0.441 |
|  | HHIP | hedgehog interacting protein | 1.25E-04 | 9.26E-03 | -0.568 |
|  | KCNJ12 | potassium inwardly rectifying channel subfamily J member 12 | 1.42E-04 | 9.66E-03 | -0.488 |
|  | MAG | myelin associated glycoprotein | 7.10E-04 | 2.11E-02 | -0.495 |
|  | MAPK3 | mitogen-activated protein kinase 3 | 3.11E-03 | 4.50E-02 | 0.238 |
|  | MME | membrane metalloendopeptidase | 4.17E-04 | 1.58E-02 | -1.105 |
|  | NGFR | nerve growth factor receptor | 2.29E-03 | 3.85E-02 | -0.765 |
|  | NRP2 | neuropilin 2 | 8.84E-05 | 8.21E-03 | 0.557 |
|  | NTF4 | neurotrophin 4 | 9.92E-05 | 8.42E-03 | 0.764 |
|  | NTRK1 | neurotrophic receptor tyrosine kinase 1 | 2.83E-04 | 1.33E-02 | -0.925 |
|  | PAK6 | p21 (RAC1) activated kinase 6 | 2.08E-03 | 3.68E-02 | 0.207 |
|  | PIK3C2B | phosphatidylinositol-4-phosphate 3-kinase catalytic subunit type 2 beta | 1.32E-05 | 3.56E-03 | -0.616 |
|  | PLXNB2 | plexin B2 | 8.46E-04 | 2.34E-02 | 0.424 |
|  | PLXNB3 | plexin B3 | 4.03E-05 | 5.55E-03 | -0.458 |
|  | PRKCD | protein kinase C delta | 2.80E-04 | 1.33E-02 | -1.24 |
|  | PRKCH | protein kinase C eta | 2.56E-04 | 1.29E-02 | -0.723 |
|  | PRKD1 | protein kinase D1 | 4.18E-04 | 1.58E-02 | -0.538 |
|  | PRKD3 | protein kinase D3 | 4.73E-04 | 1.70E-02 | -0.329 |
|  | PTCH2 | patched 2 | 2.86E-03 | 4.28E-02 | -0.497 |
|  | RAC3 | Rac family small GTPase 3 | 2.47E-03 | 3.98E-02 | 0.331 |
|  | RASD2 | RASD family member 2 | 1.93E-04 | 1.13E-02 | -1.073 |
|  | RHOD | ras homolog family member D | 2.09E-03 | 3.68E-02 | 0.846 |
|  | SEMA3B | semaphorin 3B | 3.96E-05 | 5.54E-03 | 2.399 |
|  | SEMA3E | semaphorin 3E | 4.18E-05 | 5.63E-03 | -0.647 |
|  | SEMA6A | semaphorin 6A | 2.77E-03 | 4.21E-02 | -0.388 |
|  | SEMA7A | semaphorin 7A (John Milton Hagen blood group) | 1.27E-03 | 2.87E-02 | -0.487 |
|  | SRGAP1 | SLIT-ROBO Rho GTPase activating protein 1 | 2.36E-04 | 1.23E-02 | -0.74 |
|  | TUBA1A | tubulin alpha 1a | 1.32E-03 | 2.88E-02 | 0.198 |
|  | TUBA1C | tubulin alpha 1c | 4.34E-04 | 1.60E-02 | 2.149 |
|  | TUBB2A | tubulin beta 2A class IIa | 1.96E-03 | 3.55E-02 | 0.311 |
|  | TUBB4B | tubulin beta 4B class IVb | 1.01E-05 | 3.45E-03 | 0.351 |
|  | UNC5A | unc-5 netrin receptor A | 1.43E-03 | 3.00E-02 | 0.349 |
|  | UNC5B | unc-5 netrin receptor B | 8.34E-04 | 2.32E-02 | -0.473 |
|  | WNT4 | Wnt family member 4 | 2.57E-03 | 4.06E-02 | 0.731 |
|  | WNT7B | Wnt family member 7B | 1.15E-03 | 2.73E-02 | 0.328 |
| **CRH signaling pathway** | ADCY5 | adenylate cyclase 5 | 5.24E-04 | 1.79E-02 | -0.691 |
|  | ADCY6 | adenylate cyclase 6 | 2.31E-03 | 3.88E-02 | 0.323 |
|  | BDNF | brain derived neurotrophic factor | 3.36E-03 | 4.67E-02 | 0.714 |
|  | CACNA1H | calcium voltage-gated channel subunit alpha1 H | 2.08E-03 | 3.68E-02 | -0.292 |
|  | CACNA1I | calcium voltage-gated channel subunit alpha1 I | 3.30E-04 | 1.43E-02 | -0.424 |
|  | CACNA2D1 | calcium voltage-gated channel auxiliary subunit alpha2delta 1 | 3.05E-03 | 4.45E-02 | 0.348 |
|  | CACNA2D2 | calcium voltage-gated channel auxiliary subunit alpha2delta 2 | 3.08E-04 | 1.38E-02 | -0.399 |
|  | CACNA2D3 | calcium voltage-gated channel auxiliary subunit alpha2delta 3 | 5.05E-04 | 1.75E-02 | -0.586 |
|  | CACNB2 | calcium voltage-gated channel auxiliary subunit beta 2 | 1.32E-03 | 2.88E-02 | -0.339 |
|  | CACNG4 | calcium voltage-gated channel auxiliary subunit gamma 4 | 4.74E-04 | 1.70E-02 | -0.319 |
|  | CRHR2 | corticotropin releasing hormone receptor 2 | 5.82E-05 | 6.68E-03 | 0.926 |
|  | GAD1 | glutamate decarboxylase 1 | 1.59E-03 | 3.20E-02 | -0.52 |
|  | GLI3 | GLI family zinc finger 3 | 1.97E-03 | 3.57E-02 | -0.411 |
|  | GNAI1 | G protein subunit alpha i1 | 1.48E-03 | 3.07E-02 | 0.18 |
|  | Gucy2g | guanylate cyclase 2g | 2.67E-03 | 4.15E-02 | 0.711 |
|  | MAPK3 | mitogen-activated protein kinase 3 | 3.11E-03 | 4.50E-02 | 0.238 |
|  | NPR1 | natriuretic peptide receptor 1 | 2.59E-03 | 4.08E-02 | 0.546 |
|  | NPR3 | natriuretic peptide receptor 3 | 1.13E-05 | 3.56E-03 | 1.394 |
|  | PRKCD | protein kinase C delta | 2.80E-04 | 1.33E-02 | -1.24 |
|  | PRKCH | protein kinase C eta | 2.56E-04 | 1.29E-02 | -0.723 |
|  | PRKD1 | protein kinase D1 | 4.18E-04 | 1.58E-02 | -0.538 |
|  | PRKD3 | protein kinase D3 | 4.73E-04 | 1.70E-02 | -0.329 |
|  | PTCH2 | patched 2 | 2.86E-03 | 4.28E-02 | -0.497 |
| **cAMP-mediated signaling** | ADCY5 | adenylate cyclase 5 | 5.24E-04 | 1.79E-02 | -0.691 |
|  | ADCY6 | adenylate cyclase 6 | 2.31E-03 | 3.88E-02 | 0.323 |
|  | ADORA2A | adenosine A2a receptor | 5.98E-04 | 1.93E-02 | -0.926 |
|  | ADRA2A | adrenoceptor alpha 2A | 2.31E-03 | 3.88E-02 | 0.35 |
|  | ADRA2B | adrenoceptor alpha 2B | 2.70E-03 | 4.17E-02 | -0.908 |
|  | CAMK2B | calciumcalmodulin dependent protein kinase II beta | 4.11E-03 | 5.23E-02 | -0.268 |
|  | DRD1 | dopamine receptor D1 | 1.67E-03 | 3.28E-02 | -0.826 |
|  | DRD2 | dopamine receptor D2 | 1.01E-04 | 8.43E-03 | -1.227 |
|  | GLP1R | glucagon like peptide 1 receptor | 4.26E-03 | 5.29E-02 | -0.637 |
|  | GNAI1 | G protein subunit alpha i1 | 1.48E-03 | 3.07E-02 | 0.18 |
|  | GNAL | G protein subunit alpha L | 3.26E-04 | 1.42E-02 | -0.558 |
|  | GRM3 | glutamate metabotropic receptor 3 | 4.35E-03 | 5.34E-02 | -0.284 |
|  | GRM4 | glutamate metabotropic receptor 4 | 3.62E-04 | 1.46E-02 | -0.595 |
|  | HTR1B | 5-hydroxytryptamine receptor 1B | 2.77E-04 | 1.33E-02 | -0.69 |
|  | HTR1D | 5-hydroxytryptamine receptor 1D | 3.56E-04 | 1.46E-02 | -0.736 |
|  | Htr5b | 5-hydroxytryptamine (serotonin) receptor 5B | 1.80E-03 | 3.39E-02 | 0.77 |
|  | LPAR1 | lysophosphatidic acid receptor 1 | 1.03E-03 | 2.55E-02 | -0.361 |
|  | MAPK3 | mitogen-activated protein kinase 3 | 3.11E-03 | 4.50E-02 | 0.238 |
|  | NPR3 | natriuretic peptide receptor 3 | 1.13E-05 | 3.56E-03 | 1.394 |
|  | PDE10A | phosphodiesterase 10A | 3.00E-04 | 1.37E-02 | -1.279 |
|  | PDE1A | phosphodiesterase 1A | 4.67E-03 | 5.56E-02 | 0.447 |
|  | PDE1B | phosphodiesterase 1B | 2.91E-04 | 1.35E-02 | -0.821 |
|  | PDE1C | phosphodiesterase 1C | 2.41E-03 | 3.93E-02 | -0.503 |
|  | PDE5A | phosphodiesterase 5A | 3.51E-04 | 1.45E-02 | 0.502 |
|  | PDE7B | phosphodiesterase 7B | 2.18E-04 | 1.20E-02 | -0.849 |
|  | PDE8A | phosphodiesterase 8A | 5.16E-05 | 6.40E-03 | -0.516 |
|  | PTGDR | prostaglandin D2 receptor | 4.11E-03 | 5.23E-02 | 0.689 |
|  | RAP1GAP | RAP1 GTPase activating protein | 1.73E-04 | 1.07E-02 | -0.438 |
|  | RGS4 | regulator of G protein signaling 4 | 1.31E-04 | 9.43E-03 | -0.678 |
|  | SSTR3 | somatostatin receptor 3 | 5.52E-04 | 1.86E-02 | 0.57 |
| **GABA receptor signaling** | ADCY5 | adenylate cyclase 5 | 5.24E-04 | 1.79E-02 | -0.691 |
|  | ADCY6 | adenylate cyclase 6 | 2.31E-03 | 3.88E-02 | 0.323 |
|  | AP2S1 | adaptor related protein complex 2 subunit sigma 1 | 3.48E-03 | 4.79E-02 | 0.189 |
|  | CACNA1H | calcium voltage-gated channel subunit alpha1 H | 2.08E-03 | 3.68E-02 | -0.292 |
|  | CACNA1I | calcium voltage-gated channel subunit alpha1 I | 3.30E-04 | 1.43E-02 | -0.424 |
|  | CACNA2D1 | calcium voltage-gated channel auxiliary subunit alpha2delta 1 | 3.05E-03 | 4.45E-02 | 0.348 |
|  | CACNA2D2 | calcium voltage-gated channel auxiliary subunit alpha2delta 2 | 3.08E-04 | 1.38E-02 | -0.399 |
|  | CACNA2D3 | calcium voltage-gated channel auxiliary subunit alpha2delta 3 | 5.05E-04 | 1.75E-02 | -0.586 |
|  | CACNB2 | calcium voltage-gated channel auxiliary subunit beta 2 | 1.32E-03 | 2.88E-02 | -0.339 |
|  | CACNG4 | calcium voltage-gated channel auxiliary subunit gamma 4 | 4.74E-04 | 1.70E-02 | -0.319 |
|  | GABRD | gamma-aminobutyric acid type A receptor subunit delta | 1.06E-03 | 2.60E-02 | -0.744 |
|  | GABRG3 | gamma-aminobutyric acid type A receptor subunit gamma3 | 1.37E-03 | 2.93E-02 | -0.232 |
|  | GAD1 | glutamate decarboxylase 1 | 1.59E-03 | 3.20E-02 | -0.52 |
|  | GAD2 | glutamate decarboxylase 2 | 2.20E-04 | 1.20E-02 | -0.367 |
|  | GPR37 | G protein-coupled receptor 37 | 2.69E-04 | 1.32E-02 | -0.54 |
|  | KCNQ3 | potassium voltage-gated channel subfamily Q member 3 | 3.28E-03 | 4.63E-02 | -0.226 |
|  | SLC32A1 | solute carrier family 32 member 1 | 2.71E-03 | 4.18E-02 | -0.459 |

**List of genes annotated with the top five canonical signaling pathways affected in female SIR-VEH versus GRP-VEH offspring.** Ingenuity Pathway Analysis (IPA) was used to identify canonical signaling pathways affected in the amygdala of SIR-VEH female offspring relative to GRP-VEH female offspring. The table lists the top five signaling pathways and the differentially expressed genes (DEGs) annotated with each pathway. The direction of transcriptional changes is shown in terms of log2 fold changes (Log2FC). The significance is reported both in terms of false discovery rate (FDR) correction set at a 5% threshold (*q* < 0.05) and of uncorrected p value.

**Supplementary Table S7**

|  | | | **SIR-FLX over SIR-VEH** | | |
| --- | --- | --- | --- | --- | --- |
| **Pathway** | **Gene symbol** | **Gene name** | ***p*-value** | ***q-*value** | **Log2FC** |
| **G-Protein receptor signaling** | ADCY5 | adenylate cyclase 5 | 1.63E-04 | 2.19E-02 | 0.893 |
|  | ADORA1 | adenosine A1 receptor | 4.53E-03 | 1.20E-01 | 0.319 |
|  | ADORA2A | adenosine A2a receptor | 2.13E-03 | 8.24E-02 | 1.067 |
|  | ADRA2B | adrenoceptor alpha 2B | 2.07E-03 | 8.19E-02 | 1.192 |
|  | CAMK4 | calciumcalmodulin dependent protein kinase IV | 8.52E-05 | 1.92E-02 | 0.703 |
|  | CAMK2B | calciumcalmodulin dependent protein kinase II beta | 1.85E-03 | 7.63E-02 | 0.455 |
|  | CHRM4 | cholinergic receptor muscarinic 4 | 4.26E-03 | 1.16E-01 | 0.54 |
|  | DRD1 | dopamine receptor D1 | 2.86E-03 | 9.80E-02 | 0.999 |
|  | DRD2 | dopamine receptor D2 | 5.43E-05 | 1.53E-02 | 1.452 |
|  | GNAL | G protein subunit alpha L | 8.72E-04 | 5.03E-02 | 0.628 |
|  | GRM4 | glutamate metabotropic receptor 4 | 2.72E-04 | 2.75E-02 | 0.853 |
|  | GRM5 | glutamate metabotropic receptor 5 | 2.85E-03 | 9.80E-02 | 0.331 |
|  | HRH3 | histamine receptor H3 | 2.08E-03 | 8.19E-02 | 0.475 |
|  | HTR6 | 5-hydroxytryptamine receptor 6 | 3.22E-03 | 1.03E-01 | 0.512 |
|  | HTR1B | 5-hydroxytryptamine receptor 1B | 1.05E-03 | 5.53E-02 | 0.799 |
|  | HTR1D | 5-hydroxytryptamine receptor 1D | 9.44E-04 | 5.29E-02 | 0.786 |
|  | HTR2A | 5-hydroxytryptamine receptor 2A | 4.65E-03 | 1.21E-01 | 0.646 |
|  | HTR2C | 5-hydroxytryptamine receptor 2C | 2.19E-04 | 2.51E-02 | -0.606 |
|  | NPR3 | natriuretic peptide receptor 3 | 1.06E-03 | 5.53E-02 | -1.232 |
|  | PDE10A | phosphodiesterase 10A | 1.34E-04 | 2.12E-02 | 1.585 |
|  | PDE1B | phosphodiesterase 1B | 2.00E-03 | 8.05E-02 | 0.943 |
|  | PDE7B | phosphodiesterase 7B | 1.80E-04 | 2.37E-02 | 1.015 |
|  | PLCB1 | phospholipase C beta 1 | 7.68E-04 | 4.71E-02 | 0.449 |
|  | PRKCB | protein kinase C beta | 2.71E-04 | 2.75E-02 | 0.523 |
|  | RAP1GAP | RAP1 GTPase activating protein | 1.02E-03 | 5.53E-02 | 0.497 |
|  | RASD2 | RASD family member 2 | 2.06E-04 | 2.50E-02 | 1.289 |
| **Synaptic LTD** | CACNA1I | calcium voltage-gated channel subunit alpha1 I | 3.57E-03 | 1.08E-01 | 0.753 |
|  | CACNA2D3 | calcium voltage-gated channel auxiliary subunit alpha2delta 3 | 2.02E-03 | 8.09E-02 | 0.661 |
|  | CACNB2 | calcium voltage-gated channel auxiliary subunit beta 2 | 2.91E-03 | 9.83E-02 | 0.467 |
|  | CACNB4 | calcium voltage-gated channel auxiliary subunit beta 4 | 1.01E-03 | 5.51E-02 | 0.484 |
|  | CACNG4 | calcium voltage-gated channel auxiliary subunit gamma 4 | 3.67E-03 | 1.09E-01 | 0.51 |
|  | GNAL | G protein subunit alpha L | 8.72E-04 | 5.03E-02 | 0.628 |
|  | GRM4 | glutamate metabotropic receptor 4 | 2.72E-04 | 2.75E-02 | 0.853 |
|  | GRM5 | glutamate metabotropic receptor 5 | 2.85E-03 | 9.80E-02 | 0.331 |
|  | ITPR1 | inositol 1,4,5-trisphosphate receptor type 1 | 2.21E-04 | 2.51E-02 | 0.78 |
|  | NPR1 | natriuretic peptide receptor 1 | 2.67E-04 | 2.75E-02 | -1.231 |
|  | NPR3 | natriuretic peptide receptor 3 | 1.06E-03 | 5.53E-02 | -1.232 |
|  | PLA2G5 | phospholipase A2 group V | 3.08E-04 | 2.82E-02 | -1.908 |
|  | PLCB1 | phospholipase C beta 1 | 7.68E-04 | 4.71E-02 | 0.449 |
|  | PRKCB | protein kinase C beta | 2.71E-04 | 2.75E-02 | 0.523 |
|  | PRKCH | protein kinase C eta | 2.22E-03 | 8.34E-02 | 0.84 |
|  | PRKD1 | protein kinase D1 | 1.81E-03 | 7.54E-02 | 0.589 |
|  | PRKG2 | protein kinase cGMP-dependent 2 | 4.75E-03 | 1.23E-01 | -0.465 |
|  | RASD2 | RASD family member 2 | 2.06E-04 | 2.50E-02 | 1.289 |
|  | RYR3 | ryanodine receptor 3 | 2.38E-03 | 8.65E-02 | 0.718 |
| **Opioid signaling pathway** | ADCY5 | adenylate cyclase 5 | 1.63E-04 | 2.19E-02 | 0.893 |
|  | CACNA1I | calcium voltage-gated channel subunit alpha1 I | 3.57E-03 | 1.08E-01 | 0.753 |
|  | CACNA2D3 | calcium voltage-gated channel auxiliary subunit alpha2delta 3 | 2.02E-03 | 8.09E-02 | 0.661 |
|  | CACNB2 | calcium voltage-gated channel auxiliary subunit beta 2 | 2.91E-03 | 9.83E-02 | 0.467 |
|  | CACNB4 | calcium voltage-gated channel auxiliary subunit beta 4 | 1.01E-03 | 5.51E-02 | 0.484 |
|  | CACNG4 | calcium voltage-gated channel auxiliary subunit gamma 4 | 3.67E-03 | 1.09E-01 | 0.51 |
|  | CAMK4 | calciumcalmodulin dependent protein kinase IV | 8.52E-05 | 1.92E-02 | 0.703 |
|  | CAMK2B | calciumcalmodulin dependent protein kinase II beta | 1.85E-03 | 7.63E-02 | 0.455 |
|  | GNAL | G protein subunit alpha L | 8.72E-04 | 5.03E-02 | 0.628 |
|  | GNG7 | G protein subunit gamma 7 | 1.66E-03 | 7.23E-02 | 0.98 |
|  | ITPR1 | inositol 1,4,5-trisphosphate receptor type 1 | 2.21E-04 | 2.51E-02 | 0.78 |
|  | MAPK15 | mitogen-activated protein kinase 15 | 3.50E-04 | 3.00E-02 | -1.338 |
|  | PDE1B | phosphodiesterase 1B | 2.00E-03 | 8.05E-02 | 0.943 |
|  | PLCB1 | phospholipase C beta 1 | 7.68E-04 | 4.71E-02 | 0.449 |
|  | PRKCB | protein kinase C beta | 2.71E-04 | 2.75E-02 | 0.523 |
|  | PRKCH | protein kinase C eta | 2.22E-03 | 8.34E-02 | 0.84 |
|  | PRKD1 | protein kinase D1 | 1.81E-03 | 7.54E-02 | 0.589 |
|  | RASD2 | RASD family member 2 | 2.06E-04 | 2.50E-02 | 1.289 |
|  | RGS8 | regulator of G protein signaling 8 | 5.80E-04 | 4.14E-02 | 0.56 |
|  | RGS9 | regulator of G protein signaling 9 | 3.83E-04 | 3.17E-02 | 1.366 |
|  | RPS6KA5 | ribosomal protein S6 kinase A5 | 1.95E-03 | 7.91E-02 | 0.477 |
|  | RYR3 | ryanodine receptor 3 | 2.38E-03 | 8.65E-02 | 0.718 |
| **cAMP-mediated signaling** | ADCY5 | adenylate cyclase 5 | 1.63E-04 | 2.19E-02 | 0.893 |
|  | ADORA1 | adenosine A1 receptor | 4.53E-03 | 1.20E-01 | 0.319 |
|  | ADORA2A | adenosine A2a receptor | 2.13E-03 | 8.24E-02 | 1.067 |
|  | ADRA2B | adrenoceptor alpha 2B | 2.07E-03 | 8.19E-02 | 1.192 |
|  | AKAP5 | A-kinase anchoring protein 5 | 4.34E-03 | 1.18E-01 | 0.525 |
|  | CAMK4 | calciumcalmodulin dependent protein kinase IV | 8.52E-05 | 1.92E-02 | 0.703 |
|  | CAMK2B | calciumcalmodulin dependent protein kinase II beta | 1.85E-03 | 7.63E-02 | 0.455 |
|  | CHRM4 | cholinergic receptor muscarinic 4 | 4.26E-03 | 1.16E-01 | 0.54 |
|  | DRD1 | dopamine receptor D1 | 2.86E-03 | 9.80E-02 | 0.999 |
|  | DRD2 | dopamine receptor D2 | 5.43E-05 | 1.53E-02 | 1.452 |
|  | GNAL | G protein subunit alpha L | 8.72E-04 | 5.03E-02 | 0.628 |
|  | GRM4 | glutamate metabotropic receptor 4 | 2.72E-04 | 2.75E-02 | 0.853 |
|  | HRH3 | histamine receptor H3 | 2.08E-03 | 8.19E-02 | 0.475 |
|  | HTR6 | 5-hydroxytryptamine receptor 6 | 3.22E-03 | 1.03E-01 | 0.512 |
|  | HTR1B | 5-hydroxytryptamine receptor 1B | 1.05E-03 | 5.53E-02 | 0.799 |
|  | HTR1D | 5-hydroxytryptamine receptor 1D | 9.44E-04 | 5.29E-02 | 0.786 |
|  | NPR3 | natriuretic peptide receptor 3 | 1.06E-03 | 5.53E-02 | -1.232 |
|  | PDE10A | phosphodiesterase 10A | 1.34E-04 | 2.12E-02 | 1.585 |
|  | PDE1B | phosphodiesterase 1B | 2.00E-03 | 8.05E-02 | 0.943 |
|  | PDE7B | phosphodiesterase 7B | 1.80E-04 | 2.37E-02 | 1.015 |
|  | RAP1GAP | RAP1 GTPase activating protein | 1.02E-03 | 5.53E-02 | 0.497 |
| **CRH signaling** | ADCY5 | adenylate cyclase 5 | 1.63E-04 | 2.19E-02 | 0.893 |
|  | CACNA1I | calcium voltage-gated channel subunit alpha1 I | 3.57E-03 | 1.08E-01 | 0.753 |
|  | CACNA2D3 | calcium voltage-gated channel auxiliary subunit alpha2delta 3 | 2.02E-03 | 8.09E-02 | 0.661 |
|  | CACNB2 | calcium voltage-gated channel auxiliary subunit beta 2 | 2.91E-03 | 9.83E-02 | 0.467 |
|  | CACNB4 | calcium voltage-gated channel auxiliary subunit beta 4 | 1.01E-03 | 5.51E-02 | 0.484 |
|  | CACNG4 | calcium voltage-gated channel auxiliary subunit gamma 4 | 3.67E-03 | 1.09E-01 | 0.51 |
|  | CAMK4 | calciumcalmodulin dependent protein kinase IV | 8.52E-05 | 1.92E-02 | 0.703 |
|  | CRHR2 | corticotropin releasing hormone receptor 2 | 1.31E-03 | 6.17E-02 | -0.896 |
|  | ITPR1 | inositol 1,4,5-trisphosphate receptor type 1 | 2.21E-04 | 2.51E-02 | 0.78 |
|  | KRT1 | keratin 1 | 3.14E-03 | 1.01E-01 | -0.695 |
|  | NPR1 | natriuretic peptide receptor 1 | 2.67E-04 | 2.75E-02 | -1.231 |
|  | NPR3 | natriuretic peptide receptor 3 | 1.06E-03 | 5.53E-02 | -1.232 |
|  | PRKCB | protein kinase C beta | 2.71E-04 | 2.75E-02 | 0.523 |
|  | PRKCH | protein kinase C eta | 2.22E-03 | 8.34E-02 | 0.84 |
|  | PRKD1 | protein kinase D1 | 1.81E-03 | 7.54E-02 | 0.589 |
|  | UCN3 | urocortin 3 | 3.06E-03 | 1.01E-01 | -2.093 |

**List of genes annotated with the top five canonical signaling pathways affected in female SIR-FLX versus SIR-VEH offspring.** Ingenuity Pathway Analysis (IPA) was used to identify canonical signaling pathways affected in the amygdala of SIR-FLX female offspring relative to SIR-VEH female offspring. The table lists the top five signaling pathways and the differentially expressed genes (DEGs) annotated with each pathway. The direction of transcriptional changes is shown in terms of log2 fold changes (Log2FC). The significance is reported both in terms of false discovery rate (FDR) correction set at a 5% threshold (*q* < 0.05) and of uncorrected p value.

**Supplementary Figure S9**

**Heatmaps of individual genes annotated with the top 15 ‘Disease and Functions’ modules identified as being different by Ingenuity Pathway Analysis (IPA).** For either sex, the heatmaps provides comparisons between SIR-VEH and SIR-FLX offspring**.** The color-coded key denotes the level of upregulation (blue tones) or downregulation (magenta tones) in the amygdala of SIR-VEH and SIR-FLX male and female offspring.

**Supplementary References**

1 Meyer, U., Spoerri, E., Yee, B. K., Schwarz, M. J. & Feldon, J. Evaluating early preventive antipsychotic and antidepressant drug treatment in an infection-based neurodevelopmental mouse model of schizophrenia. *Schizophr Bull* **36**, 607-623, doi:10.1093/schbul/sbn131 (2010).

2 Meyer, U., Feldon, J., Schedlowski, M. & Yee, B. K. Towards an immuno-precipitated neurodevelopmental animal model of schizophrenia. *Neurosci Biobehav Rev* **29**, 913-947, doi:10.1016/j.neubiorev.2004.10.012 (2005).

3 Mueller, F. S., Polesel, M., Richetto, J., Meyer, U. & Weber-Stadlbauer, U. Mouse models of maternal immune activation: Mind your caging system! *Brain Behav Immun* **73**, 643-660, doi:10.1016/j.bbi.2018.07.014 (2018).

4 Scarborough, J. *et al.* Preclinical validation of the micropipette-guided drug administration (MDA) method in the maternal immune activation model of neurodevelopmental disorders. *Brain Behav Immun* **88**, 461-470, doi:10.1016/j.bbi.2020.04.015 (2020).

5 Meyer, U., Feldon, J. & Fatemi, S. H. In-vivo rodent models for the experimental investigation of prenatal immune activation effects in neurodevelopmental brain disorders. *Neurosci Biobehav Rev* **33**, 1061-1079, doi:10.1016/j.neubiorev.2009.05.001 (2009).

6 Zorrilla, E. P. Multiparous species present problems (and possibilities) to developmentalists. *Dev Psychobiol* **30**, 141-150, doi:10.1002/(sici)1098-2302(199703)30:2<141::aid-dev5>3.0.co;2-q (1997).

7 Weber-Stadlbauer, U. *et al.* Transgenerational transmission and modification of pathological traits induced by prenatal immune activation. *Mol Psychiatry* **22**, 102-112, doi:10.1038/mp.2016.41 (2017).

8 Meyer, U. *et al.* Adult behavioral and pharmacological dysfunctions following disruption of the fetal brain balance between pro-inflammatory and IL-10-mediated anti-inflammatory signaling. *Mol Psychiatry* **13**, 208-221, doi:10.1038/sj.mp.4002042 (2008).

9 Meyer, U. *et al.* Relative prenatal and postnatal maternal contributions to schizophrenia-related neurochemical dysfunction after in utero immune challenge. *Neuropsychopharmacology* **33**, 441-456, doi:10.1038/sj.npp.1301413 (2008).

10 Meyer, U., Nyffeler, M., Yee, B. K., Knuesel, I. & Feldon, J. Adult brain and behavioral pathological markers of prenatal immune challenge during early/middle and late fetal development in mice. *Brain Behav Immun* **22**, 469-486, doi:10.1016/j.bbi.2007.09.012 (2008).

11 Nichols, D. J. & Chevins, P. F. Effects of housing on corticosterone rhythm and stress responses in female mice. *Physiol Behav* **27**, 1-5, doi:10.1016/0031-9384(81)90291-2 (1981).

12 Ekambaram, G., Sampath Kumar, S. K. & Joseph, L. D. Comparative Study on the Estimation of Estrous Cycle in Mice by Visual and Vaginal Lavage Method. *J Clin Diagn Res* **11**, AC05-AC07, doi:10.7860/JCDR/2017/23977.9148 (2017).

13 Hatakeyama, M. *et al.* SUSHI: an exquisite recipe for fully documented, reproducible and reusable NGS data analysis. *BMC Bioinformatics* **17**, 228, doi:10.1186/s12859-016-1104-8 (2016).

14 Qi, W., Schlapbach, R. & Rehrauer, H. RNA-Seq Data Analysis: From Raw Data Quality Control to Differential Expression Analysis. *Methods Mol Biol* **1669**, 295-307, doi:10.1007/978-1-4939-7286-9_23 (2017).

15 Richetto, J. *et al.* Behavioral effects of the benzodiazepine-positive allosteric modulator SH-053-2'F-S-CH(3) in an immune-mediated neurodevelopmental disruption model. *Int J Neuropsychopharmacol* **18**, doi:10.1093/ijnp/pyu055 (2015).

16 Livak, K. J. & Schmittgen, T. D. Analysis of relative gene expression data using real-time quantitative PCR and the 2(-Delta Delta C(T)) Method. *Methods* **25**, 402-408, doi:10.1006/meth.2001.1262 (2001).
